# Isobavachalcone ameliorates Alzheimer disease pathology by autophagy-mediated clearance of amyloid beta and inhibition of NLRP3 inflammasome in primary astrocytes and 5x-FAD mice

**DOI:** 10.3389/fphar.2025.1525364

**Published:** 2025-03-20

**Authors:** Dilpreet Kour, Parul Khajuria, Kuhu Sharma, Alpa Sharma, Ankita Sharma, Syed Mudassir Ali, Priya Wazir, P. Ramajayan, Sanghapal D. Sawant, Utpal Nandi, Zabeer Ahmed, Ajay Kumar

**Affiliations:** ^1^ Pharmacology Division, CSIR-Indian Institute of Integrative Medicine, Jammu, India; ^2^ Academy of Scientific and Innovative Research (AcSIR), Ghaziabad, India; ^3^ Natural Products and Medicinal Chemistry Division, CSIR-Indian Institute of Integrative Medicine, Jammu, India; ^4^ Organic Chemsitry Division, CSIR-National Chemical Laboratory, Pune, India

**Keywords:** Alzheimer disease, amyloid beta, autophagy, isobavachalcone, neuroinflammation, NLRP3 inflammasome

## Abstract

**Background and Aim:**

Alzheimer’s disease (AD) progresses with Aβ plaque deposition and neuroinflammation. Given the complexity of AD pathology, single-target therapies have frequently failed in clinical trials. We hypothesized that a multitarget approach could yield better therapeutic outcomes. To this end, we identified isobavachalcone (IBC), a natural compound with dual pharmacological activity in reducing Aβ plaques and neuroinflammation.

**Experimental Procedure:**

Primary astrocytes were isolated from 3 to 4 days old C57BL/6J mice pups for *in-vitro* assays, while *in-vivo* studies were conducted on 5x-FAD mice. Protein alterations were evaluated using ELISA, western blotting, immunocytochemistry, and immunohistochemistry. Behavioral analyses included the radial arm maze, open field, and rotarod tests. Data from all *in vitro* and *in vivo* experiments were analyzed by using one-way ANOVA and *post-hoc* Bonferroni tests.

**Results:**

*In-vitro* analyses in astrocytes demonstrated that IBC at 5 and 10 μM concentrations induce AMPK phosphorylation through CAMKK2, promoting autophagy and inhibiting the NLRP3 inflammasome in primary astrocytes. IBC-treated astrocytes exhibited significant clearance of extracellular amyloid beta. Mechanistic studies highlighted autophagy as a key factor in reducing both NLRP3 inflammasome activity and Aβ levels. Two months of treatment of 5x-FAD mice with IBC at 25 and 50 mg/kg significantly improved cognitive functions, as evidenced by enhanced memory and motor performance in behavioral tests. Subsequent brain tissue analysis revealed that IBC upregulated autophagic proteins to reduce the brain’s amyloid beta levels, resulting in decreased expression of neuroinflammation markers.

**Conclusion:**

IBC effectively ameliorates AD pathology through autophagy-mediated clearance of Aβ and suppressing neuroinflammation in 5x-FAD mice.

## Graphical Abstract

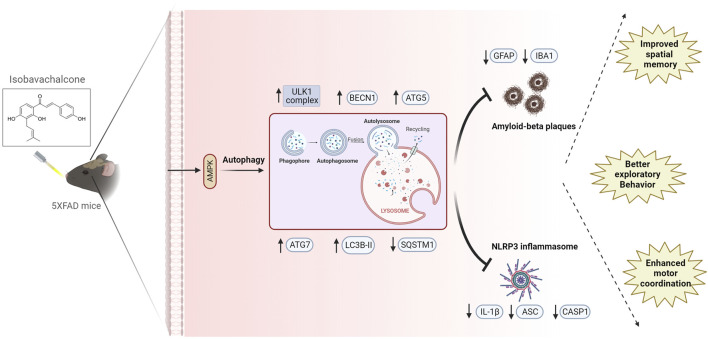

## Introduction

Alzheimer disease (AD) is the most frequently occurring age-related neurodegenerative disorder. AD symptoms include cognitive decline and behavioral and psychological instability (Kumar et al., 2023). AD progresses with unregulated deposition of amyloid-beta (Aβ) protein plaques, which facilitates the formation of neurofibrillary tangles (NFTs) by causing tau protein hyperphosphorylation. The Aβ hypothesis nominates Aβ as the paramount player in AD progression (Hardy and Allsop, 1991). Additional pathological features of AD include tau protein hyperphosphorylation, activated glial cells, enlarged endosomes, synaptic dysfunction, and loss of neurons and neural networks (Knopman et al., 2021). The Aβ and tau anomalies in the brain lead to the innate immune response by the activated glial cells, causing neuroinflammation (Al-Ghraiybah et al., 2022; Rajmohan and Reddy, 2017). The aberrant activation of NOD-like receptor family pyrin domain containing 3 (NLRP3) inflammasome by Aβ plaques, tau fibrils, and neuronal death is also the major significant contributor to AD pathogenesis (Van Zeller et al., 2021).

NLRP3 inflammasome is a component of innate immunity activated in response to varied endogenous and exogenous ligands. NLRP3 inflammasome complex comprises three proteins: sensor NLRP3, adaptor ASC (apoptosis-associated speck-like protein containing a caspase recruitment domain) and protease pro-caspase-1 (Kelley et al., 2019). Assembly formation of NLRP3 inflammasome completes with self-activation of pro-caspase 1 into effector caspase-1 (CASP1). Caspase-1 causes cleavage and secretion of pro-inflammatory cytokines, interleukin-1β (IL-1β) and interleukin-18 (IL-18). IL-1β secreted upon NLRP3 inflammasome activation produces detrimental effects on AD patients (Liang et al., 2022). These secreted inflammatory cytokines result in neuronal degeneration, decreased Aβ clearance, and expedited tau tangle formation. It can mediate a positive feedback loop, which amplifies disease symptoms (Ising et al., 2019; Luciunaite et al., 2020; Saresella et al., 2016).

The dysfunctions in the autophagy-lysosomal pathway, the major cellular pathway for clearance of accumulated damaged proteins and organelles, are conducive to the progression of AD (Nilsson and Saido, 2014; Lee et al., 2022). NLRP3 inflammasome activation can be regulated by inducing macroautophagy/autophagy in the glial cells (Mishra et al., 2021; Wen et al., 2019). By eliminating the sensor NLRP3 ligands like reactive oxygen species (ROS) generating damaged mitochondria and particulate matter from the cell, autophagy is known for regulating NLRP3 inflammasome over-activation. Autophagy regulates extracellular accumulation of Aβ, and impairment in autophagy enhances neurodegeneration and cognitive dysfunctions (Nilsson et al., 2013). Enhancing autophagy can effectively reduce Aβ burden from the brain (Wani et al., 2021; Wani et al., 2019).

The complexity and heterogeneity of AD etiology drive us to assess multi target approach for AD management in mouse model of disease. Inducing autophagy can potentially inhibit NLRP3 inflammasome and Aβ plaque formation. It can be a promising therapeutic target for AD. Despite being present in large numbers in the brain and their recently emerging role in neurodegeneration, astrocytes have not been explored much as a target in AD drug discovery. Astrocytes regulate synaptic transmissions, protect, and support neurons, and manage toxins infiltration along with microglia, etc. (Sofroniew and Vinters, 2010). Particularly in AD, the early physiological dysfunction of astrocytes to clear Aβ debris and their pathological involvement in neuroinflammation plays a crucial role in cognitive impairments (Garwood et al., 2017; Gonzalez-Reyes et al., 2017). Hence, we used astrocytes as our *in-vitro* model system.

**FIGURE 1:**
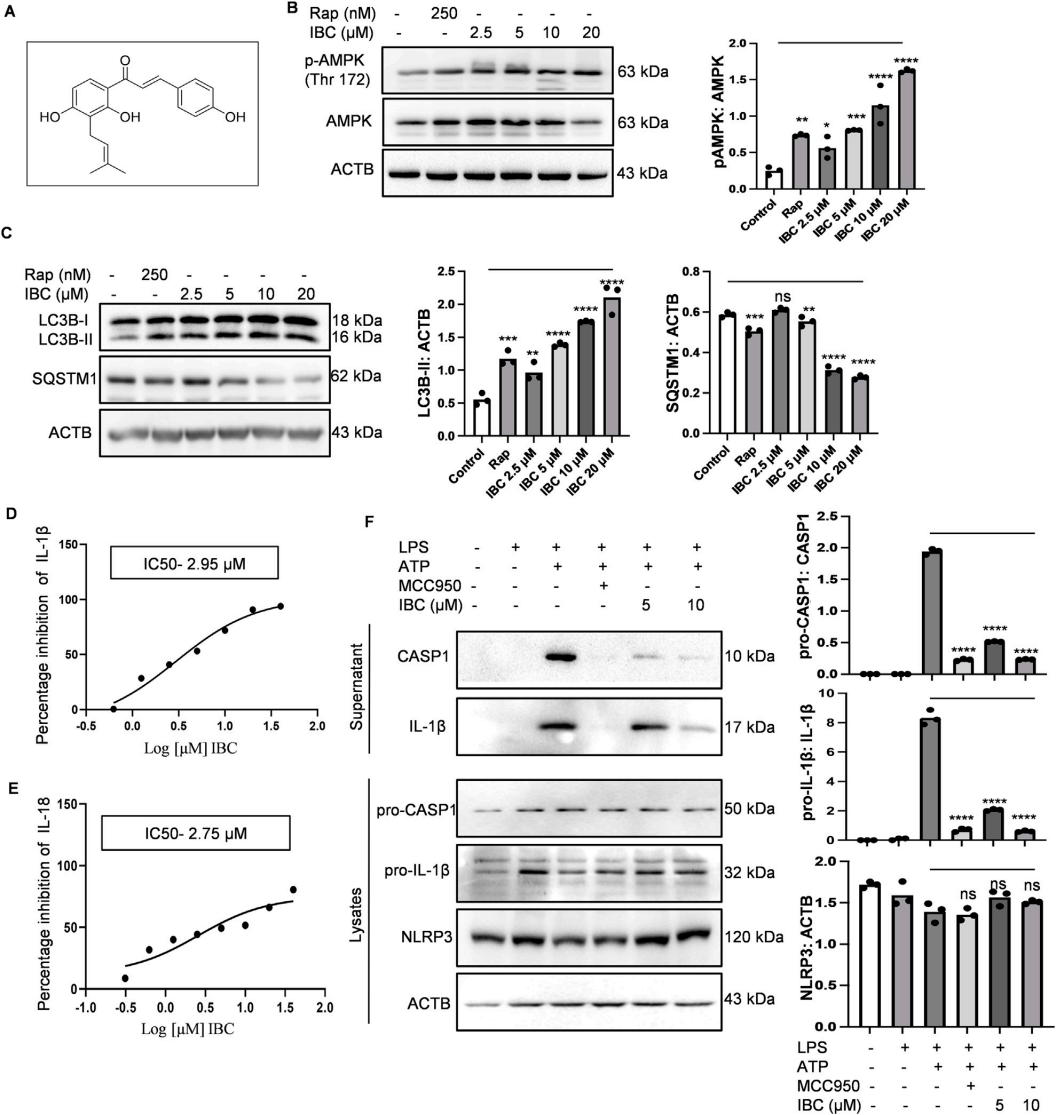
IBC induces autophagy and inhibits NLRP3 inflammasome in primary astrocytes. **(A)** Structure of IBC. Primary astrocytes were treated with IBC or rapamycin (Rap) for 24 h and levels of autophagy pathway proteins were analyzed through immunoblotting. **(B)** Immunoblot and densitometry analysis depicting the effect of IBC on AMPK phosphorylation. **(C)** Immunoblots and densitometry analysis of autophagy marker proteins, LC3B-II and SQSTM1. For NLRP3 inflammasome activity, primary astrocytes were treated with microglial conditional media and primed with LPS (1 μg/ml) for 4 h. IBC or MCC950 (100 nM) was given to washed cells for 1 h followed by ATP 5 mM stimulation for 30 min **(D)** IC50 of IBC for IL-1β, **(E)** IC50 of IBC for IL-18. **(F)** Effect of IBC on NLRP3 inflammasome complex protein. Densitometry analyses were done using ImageJ software. The statistical significance of data was calculated through one-way ANOVA analysis, followed by the Bonferroni test as *post hoc* (n = 3). p values ****p < 0.0001, ***p < 0.001, **p < 0.01, *p < 0.05. Rap, Rapamycin; IBC, Isobavachalcone; ACTB, Actin B; AMPK, 5′ adenine monophosphate-activated protein kinase; LC3, light chain 3; SQSTM1, Sequestosome 1; LPS, lipopolysaccharide; ATP, 5′ adenosine triphosphate; CASP1, caspase1; IL-18, interleukin-18; IL-1β, interleukin-1β; NLRP3, NOD-like receptor family pyrin domain containing 3.

In this study, we aimed to see the effect of a compound that concomitantly targets two critical pathways; Aβ accumulation and NLRP3 inflammasome-induced neuroinflammation, on AD pathology. To explore it, we identified naturally occurring chalcone, isobavachalcone (IBC) as a potent inducer of autophagy. By activating AMPK (5′-adenosine monophosphate-activated protein kinase)-dependent autophagy, IBC effectively suppresses the NLRP3 inflammasome and reduces Aβ deposition. IBC (Figure 1A) can be procured from the seeds of the *Psoralea corylifolia* plant and is rich in varied pharmacological properties, including anti-inflammatory, anti-cancer, neuroprotective, and antioxidative effects (Wang et al., 2021). Our data suggests that IBC and other compounds that target multiple pathways involved in disease progression can act as effective candidates for drug development against AD.

## Materials and methods

### Chemicals and reagents

#### Chemicals

Dulbecco’s modified eagle medium (Sigma-Aldrich, D1152), RPMI-1640 (Sigma-Aldrich, R6504), Streptomycin (Sigma-Aldrich, S6501), Penicillin (Sigma-Aldrich, P3032), Triton X-100 (Sigma-Aldrich, T8787), Sodium bicarbonate (Sigma-Aldrich, S5761), Phosphate buffer saline (Sigma-Aldrich, D5652), Lipopolysaccharide (Sigma-Aldrich, L3129), ATP (Sigma-Aldrich, A6419), EDTA (Invitrogen, 15575038), CP-456773 sodium salt/MCC950 (Sigma-Aldrich, PZ0280), Fetal Bovine Serum (GIBCO, 10270106), Acrylamide (MP Biomedical, 193982), Glycine (MP Biomedical, 194825), Albumin Bovine Fraction V (MP Biomedical, 160069), Phenylmethylsulphonyl fluoride (MP Biomedical, 195381), Skimmed milk (Himedia, GRM1254), Hanks’ Balanced Salt Solution (Sigma-Aldrich, H6648), Strataclean resin (Agilent, 400714-61), bafilomycin A_1_ (Sigma-Aldrich, B1793), Rapamycin (Sigma-Aldrich, R8781), Resveratrol (Sigma-Aldrich, R5010), H_2_DCFDA (Sigma-Aldrich, D6883), DAPI (Sigma-Aldrich, D9542), glycerol (Sigma-Aldrich, G5516), Tween 20 (Sigma-Aldrich, P7949), HEPES (Sigma-Aldrich, H3375), Paraformaldehyde (Sigma-Aldrich, P6148), MTT (Sigma-Aldrich, M5655), Trizma (Sigma-Aldrich, T6066), SDS (Sigma-Aldrich, L3771), Uric acid sodium salt (Sigma-Aldrich, U2875), Suberic acid (Sigma-Aldrich, S1885), OPTI-MEM media (Gibco, 11058-021), β-Amyloid (Anaspec, AS-60479), 2X Laemmli buffer (Bio-Rad-1610737).

#### Antibodies

BECN1 (Santa Cruz Biotechnology, SC-48341, #K2217), ATG5 (Santa Cruz Biotechnology, SC-133158, #J1817), ASC (Santa Cruz Biotechnology, SC-22514, #J1315), CASP1 (Santa Cruz Biotechnology, SC-56036, #J1913), ATG7 (Santa Cruz Biotechnology, SC-33211, #J1713), BCL-2 (Santa Cruz Biotechnology, SC-7382, #G2915), LAMP1 (Santa Cruz Biotechnology, SC-20011, #J2020), HRP-linked anti-goat IgG (Santa Cruz Biotechnology, SC-2354, #K2019), Control SiRNA (Santa Cruz Biotechnology, SC-37007, #B0818), AMPK (Cell Signaling Technology, 2532S), pAMPK (Cell Signaling Technology, 2535S, #19), NLRP3 (Cell Signaling Technology, 15101S, #3), *siRNA* AMPK (Cell Signaling Technology, 6620S, #3), MTOR (Cell Signaling Technology, 2972S, #9), pMTOR (Cell Signaling Technology, 5536S, #9), pCAMKK2 (Cell Signaling Technology, 12818S, #2), pULK1 (Cell Signaling Technology, 14202S, #5), FIP200 (Cell Signaling Technology, 12436S, #1), GFAP (Cell Signaling Technology, 80788S, #2), IBA1 (Cell Signaling Technology, 17198S, #3), NeuN (Cell Signaling Technology, 12943S, #1), HRP-linked anti-rabbit IgG (Cell Signaling Technology, 7074S, #28), HRP-linked anti-mouse IgG (Cell Signaling Technology, 7076S, #38), anti-mouse IgG Alexa fluor 488 (Cell Signaling Technology, 4408S, #18), anti-mouse IgG Alexa fluor 555 (Cell Signaling Technology, 4409S, #18), anti-rabbit IgG Alexa fluor 488 (Cell Signaling Technology, 4412S, #21), anti-rabbit IgG Alexa fluor 555 (Cell Signaling Technology, 4413S, #16), (anti-mIL-1β (R & D biotechnology, AF-401-NA, #NP3417091), anti-ACTB (Sigma-Aldrich, A3854, #000016757), ANTI-LC3B-II (Sigma-Aldrich, L7543, #0000121567), Anti-SQSTM1/p62 (Sigma-Aldrich, P0067).

#### Kits and other reagents

PVDF Membrane (Millipore, ISEQ00010), ECL-kit (Millipore, WBKLS0500), Precision plus protein markers (Bio-Rad, 161-0375), Bradford reagent (Bio-Rad, 500-0006), FuGENE HD (Promega, E2313), IL- 1β ELISA kit (Invitrogen, 88-7013-88), IL- 18 ELISA kit (Invitrogen, 88-50618-88), Human Aβ42 ELISA kit (Invitrogen, KHB3441).

### Preparation, fractionation, and isolation of isobavachalcone from *Psoralea corylifolia* extracts

The seeds of the plant *Psoralea corylifolia* were purchased from the local market of Jammu, J&K, India. The starting material used is FSSAI (Food Safety and Standards Authority of India) certified with license number 13319011000193. The dried seeds (2.7 kg) of the plant were coarsely powdered by grinding and extracted with a mixture of DCM (dichloromethane): MeOH (methanol) (1:1, 15 L) at room temperature for 24 h and filtered. The marc was reextracted twice with the same solvent mixture using the same volume ratio of material to solvent. The filtrates were combined and concentrated on a rotary evaporator under reduced pressure at 40°C to yield 322.5 g crude extract with an extractive value (EV) of 11.9%.

DCM: MeOH (1:1) extract was fractionated sequentially with three different solvents, viz hexane, dichloromethane, and methanol by liquid-liquid partition chromatography to yield an enriched fraction of isobavachalcone. 300 g of the dried extract was dissolved in 1.5 L methanol and partitioned with 2 L hexane and this whole procedure was repeated two more times. After separating the upper hexane layer, 1.5 L DCM and 150 mL distilled water were added to the remaining methanolic layer for partitioning and the process was repeated two more times for maximum enrichment. All three fractions; hexane (108.4 g), DCM (119.7 g), and aqueous methanol (69.8 g) thus obtained were monitored by thin layer chromatography (TLC). TLC analysis concluded that the DCM fraction had the highest concentration of isobavachalcone followed by the hexane fraction.

Preferentially, the DCM fraction (100 g) was charged on a silica-gel column (60-120 mesh) and eluted with a gradient of *n*-hexane-ethyl acetate (95:5–50:50, v/v) which provided several subfractions. These subfractions were further analyzed by TLC using solvent system *n*-hexane/ethyl acetate (70:30). Based on the identification of isobavachalcone on TLC plates, the subfractions are pooled together and charged on silica gel column (60-120) to furnish the desired compound that is Isobavachalcone (2.1 g).

The purity of the IBC was analyzed using High-Performance Liquid Chromatography (HPLC) (Model: Prominence, Make: Shimadzu). Chromatographic separation was performed in gradient mode on a Purospher STAR RP-18 (250 × 4.6 mm, 5 μm) column. The mobile phase consisted of 0.1% formic acid in water (A) and acetonitrile (B). The flow rate was set to 1 mL/min, with a total run time of 60 min. The sample eluted at 30.3 min, and the detection was carried out at a wavelength of 364 nm. The stock solution was prepared in DMSO and subsequently diluted with methanol for analysis.

### Primary astrocytes culture

Primary astrocytes were cultured from C57BL/6J (Jax:000664) mice pups with the approval of Institutional Animal Ethics Committee (IAEC) of CSIR-Indian Institute of Integrative Medicine (IIIM), Jammu, under IAEC number 287/80/2/2022 and 305/81/8/2022. The astrocytes were isolated from the brain of 3–4 days-old mice pups and were grown in 10% FBS-supplemented DMEM media. Briefly, pups were sacrificed through decapitation and sterilized with 70% ethanol. Brain was exposed and isolated. Olfactory lobes and cerebellum were removed with fine blade. The cortical region separated was collected in incomplete media (media without fetal bovine serum) and cut into small pieces. After that, these cortices were incubated at 37°C in 0.25% trypsin for 30 min, with gentle shaking after every 10 min. Trypsin activity was neutralized with complete media and cells were collected using a 70-micron cell strainer followed by centrifugation at 300 g for 5 min. The pellet was dissolved in fresh media and cells were grown in cell culture flasks and were maintained at 37°C in a CO_2_ incubator. Microglia and oligodendrocytes were separated from the underlying astrocyte layer after 6-7 days by shaking the flask for 6 h at 240 rpm. Cultures astrocytes within 2-4 passage were used for all the experiments.

### NLRP3 inflammasome activation

Primary astrocytes were first treated with microglia conditional media to induce astrogliosis (Liddelow et al., 2017). For this, the microglia (N9) cell line, provided by Dr. Anirban Basu (National Brain Research Centre, Gurgaon, India) was cultured in RPMI-1640. N9 cells (Passage 28-35) were seeded in 24 well plate at density 0.05 × 10^6^ per well. The cells were treated with 1 μg/ml LPS, and supernatant was collected after 24 h. Seeded astrocytes were then treated with the LPS stimulated N9 cells supernatant for 24 h. Thereafter, the treatment for NLRP3 inflammasome activation was given, including priming with fresh 1 μg/ml LPS for 4 h, followed by washing and IBC or MCC950 (100 nM) treatment for 1 h in incomplete media (media without fetal bovine serum). ATP at 5 mM concentration was used as secondary signal for 30 min.

The experiment was terminated by collecting the cell supernatant.

### Cytokines measurement by ELISA

After the termination of experiments, the supernatant was collected and analyzed for levels of cytokines. For measuring IL-1β and IL-18, supernatant collected after NLRP3 inflammasome activation as mentioned above, was subjected to ELISA. For detection of IL-1β in mice cortex, mice cortices were isolated and homogenized in RIPA buffer to measure IL-1β in homogenates. The manufacturer’s protocol was followed for measuring the cytokines. The levels of measured cytokines were normalized with the total protein content of the cells. For total protein content of cell pellet lysed with lysis buffer (0.2 N NaCl and 1% Triton-X 100) was measured.

### IC50 calculation

IC50 of Isobavachalcone for IL-1β and IL-18 was calculated by using GraphPad Prism 8 software using non-linear regression analysis model. Log (inhibitor) vs. response curve was plotted.

### ASC oligomer formation

Following treatment with 5 and 10 μM IBC (1 h) and NLRP3 inflammasome activation in primary astrocytes, cells were lysed with ice-cold buffer (KCl [150 mM], HEPES-KOH [20 mM, pH 7.5], NP-40 [1%], sodium orthovanadate [1 mM], PMSF [0.1 mM] and protease inhibitor cocktail [1%]). Cells were centrifuged at 300 g for 10 min. The supernatant was collected for western blot analysis of total ASC protein. The pellet was washed with cold PBS and resuspended in 500 µL cold PBS. For the crosslinking of ASC, 2 mM of suberic acid was added to the resuspended pellet and incubated at 37°C for 30 min in water bath. After centrifugation, the supernatant was discarded, and a 2X Laemmli buffer was added to the pellet. The samples were heated at 95°C for 5 min and subjected to western blotting for analyzing ASC oligomers.

### Cell viability assay

For cell viability assay, primary astrocytes were seeded in 96 well plate and IBC treatment at different concentration was given for 24 h. For MTT assay, cells were treated with 1.25, 2.5, 5, 10, 20, 40, 80 and 100 μM of IBC, while for the SRB assay, 0.625, 1.25, 2.5, 5, 100, 20, 40, 80 and 100 μM IBC treatment was given. MTT dye at a concentration of 2.5 mg/ml was added 4 h before experiment termination. Formazan crystals thus formed were dissolved in DMSO and absorbance was measured at 570 nm.

For the SRB assay, the experiment was terminated by adding 50% chilled TCA into the wells for 1 h. Pellets were washed five times with distilled water and kept drying. Cells were then incubated with SRB dye for 30 min at RT, followed by washing with 1% acetic acid thrice. After drying, pellets were dissolved in 10 mM Tris-HCl (pH-10.5) and absorbance was measured at 540 nm.

### Immunoblotting

Levels of cleaved IL-1β and caspase-1 were analyzed in cellular supernatant. The supernatant collected after the end of the experiment was incubated with StartaClean resin for 1 h at 4°C. Samples were kept on rotation so that the resin could bind properly with proteins. The concentrated resin pellet was obtained by centrifuging samples at 300 g for 10 min 2X Laemmli buffer was added to the resin pellet, heated at 95°C for 10 min, and subjected to western blotting.

Cellular lysates were prepared using radio immuno precipitation assay (RIPA) buffer for protein analysis. Briefly, the cell pellet was dissolved in RIPA buffer containing sodium fluoride, sodium orthovanadate, phenyl methyl sulfonyl fluoride (PMSF) and 1% protease inhibitor cocktail for 1 h with vertexing sample every 15 min. The supernatant was collected after high-speed centrifugation of samples for 20 min at 4°C. For analysis of proteins in mice hippocampi, the hippocampi isolated from mice brains was immediately washed with saline and stored in liquid nitrogen. For lysates preparation, tissue was homogenized in RIPA buffer substituted with sodium fluoride, sodium orthovanadate, phenyl methyl sulfonyl fluoride and 1% protease inhibitor cocktail. Total protein was measured using Bradford assay. Proteins were separated using SDS-PAGE and then transferred from gel to PVDF membrane. The protein of interest was labeled with a specific primary antibody overnight at 4°C. Further, the blot was incubated with corresponding HRP-conjugated secondary antibody for 1 h at room temperature. The band were visualized in the Genaxy Chemi Doc imaging system (Make: Syngene, Maryland, USA; Model: G: BOX, XT-4). Densitometry analysis of all the immunoblots was performed by using ImageJ software. Band density of proteins was normalized with β-actin. The phosphorylated proteins were normalized with their total forms.

### Fluorescence microscopy

Cells were seeded on coverslips in 6 well plates. After treatments, cells were fixed with 4% paraformaldehyde (PFA) for 15 min and permeabilized with 0.1% Triton-X 100 for 7 min. Blocking was done for 30 min with 2% BSA, followed by overnight incubation with primary antibody. Alexa fluor conjugated secondary antibody incubation was given to cells for 1 h for labeling. The nucleus was stained with 4,6-diamidino-2-phenylindole (DAPI) (1 μg/mL) for 10 min. Following staining, the coverslips were mounted on slides using mounting media (PBS and glycerol at a ratio of 1:9). Cells were washed thrice with PBS after each incubation. The images were acquired in a CQ1 high-throughput imaging system.

### Aβ clearance assay

Aβ_42_-HiLyte fluor488 peptide was prepared following the manufacturer’s protocol (Anaspec Inc.). Primary astrocytes seeded on coverslips were treated with IBC (10 μM) or rapamycin (24 h). After 12 h, 2 μg/ml fluorochrome tagged (Hilyte fluor488) Aβ_42_ protein was added to cells for another 12 h. Bafilomycin A_1_ was given 3 h prior to experiment termination. After the end of the assay, cells were washed with PBS and cells were fixed with 4% PFA, permeabilized with Triton-X 100, and the nucleus was stained with DAPI, as mentioned above. Similarly, slides were prepared, and images were taken and analyzed in the CQ1 high throughput imaging system.

### Transfection with *siAMPK*

AMPK expression was knocked down from the cells by using *siRNA* against AMPK using FuGENE HD for 24 h. Primary astrocytes seeded in 6 well plates were incubated in OPTI-MEM media for 30 min prior to transfection. The transfection mixture was prepared and kept in sterile condition for 20 min before being added to the cells. Following this, cells were treated for NLRP3 inflammasome activation as described above.

### *In-vivo* experiments

All animal experiments were conducted with the approval of the Institutional Animal Ethics Committee of CSIR-IIIM, Jammu (IAEC No. 305/81/8/2022), adhering to the ethical guidelines of CCSEA (https://ccsea.gov.in). The mice were housed in IVCs (Individually ventilated cages) under controlled environmental conditions with a temperature 25°C ± 2°C, Relative Humidity 50%-60%, and a light cycle of 12 h light/dark. Gamma-irradiated feed and autoclaved RO water were provided as *ad libitum* to all experimental animals. At the end of experiments, animals were humanely euthanized by CO_2_ asphyxiation.

### Pharmacokinetic analysis

C57BL/6J (Jax:000664) male mice were used for pharmacokinetic analysis of IBC. Mice were randomly sorted into 9 groups (n = 5). 30 mg/kg oral dose of IBC (1% DMSO +70% PEG-200 + 29% water) was given to mice. Blood or brain samples were collected at 0, 0.25, 0.5, 1, 2, 4, 6, 8 and 24 h. The plasma (100 µL) sample was processed with ethyl acetate (200 µL) containing IS (150 ng/mL) and centrifuged at 300 g for 10 min. The organic layer was separated and dried in a vacuum, reconstituted with acetonitrile, and analyzed by LC-MS/MS [SRM transition of IBC and IS (Phenacetin) is 325.2 & gt; 149.0 and 180.2 & gt; 110.2 in Positive mode]. Brain homogenate was prepared using the weighted amount of brain tissue in water at the concentration level of 350 mg/mL and processed similarly to plasma samples.

### 5x-FAD transgenic mice

Hemizygous 5x-FAD transgenic mice (B6.Cg-Tg(APPSwFlLon, PSEN1*M146L*L286V) 6799Vas/Mmjax; MMRRC Strain: 034848-JAX) on congenic C57BL/6J genetic background were used for experiments. The 5x-FAD mice were genotyped using APP-specific qPCR probes to detect the transgene as per the protocol of Jackson Laboratory. Six months old 5x-FAD mice were randomly divided into three groups (n = 5), based on their body weights, with three males and two females in each group. We used mixed-cohort of males and females for our study to exclude any gender biased effect of IBC. Also, AD symptoms are more pronounced in females than in males (Aggarwal and Mielke, 2023), making it possible that males and females respond differently to the drug and treatment. Considering this, it is important to show effect of IBC on both sexes. At this age, Aβ deposition and neuroinflammation has already progressed in these mice. We have used IBC as a curative drug rather than a preventive drug. Prior to the initiation of the study, all the animals were acclimatized for 1 week under standard laboratory conditions; animals were drug naive with no prior procedures performed. All testing were performed from 1 to 4 p.m. IBC was orally (OD) given to two groups at different doses, 25 mg/kg (1% DMSO, 2% Tween 80, 19% PEG200, and 78% distilled water) and 50 mg/kg (1% DMSO, 5% Tween 80, 19% PEG200% and 75% distilled water) using oral gavage. 5x-FAD control mice were administered with vehicle (1% DMSO, 5% Tween 80, 19% PEG200% and 75% distilled water). For behavioral and Immunohistochemistry assays, experimenters were blinded to animal identity while carrying out the procedures.

### Behavioral analysis

To check the effect of IBC on the behavior of 5x-FAD mice, open field test (OFT), rotarod (Make; Ugo Basile, rota-rod for mice 7650) and radial arm maze (Make; Ugo Basile) tests were performed. All behavioral assays and testing were performed by an experimenter blinded to the treatment groups. To check the effect of IBC on motor coordination and balancing rotarod test was performed. After acclimatizing mice with rotarod for 5 days, their latency to fall was calculated on the day of analysis. The given final values are the mean of three independent attempts of each mouse. OFT was performed to assess exploratory and locomotor activity in a white box (45 × 60 cm). Distance traveled, speed, time spent in corner and center zones and mobile episodes were recorded through an automated video tracking system using AnyMaze software. To check the effect of IBC on the spatial memory of mice, the eight-armed radial arm maze test was done. The mice were acclimatized to the maze for 5 days before the experiment. Each mouse was trained to navigate the maze for five consecutive days. The mouse was placed on the end of one arm (entry arm). For spatial memory assessment, a food reward (100 mg of butter cookies) was placed in the selected arm, which was considered as entry arm. The movement of mice was tracked with the help of automated AnyMaze software connected with a tracking camera and various memory retention parameters were assessed.

### Immunohistochemistry

Mice’s hippocampus tissue was fixed in 10% formalin. Dehydrated tissue slices were made and placed on slides. For analysis, tissue was rehydrated following subsequent incubation in xylene, xylene: ethanol, 100% ethanol, 90% ethanol, 70% and 50% ethanol. After tissue rehydration, cells were permeabilized using 0.4% Triton-X100 for 10 min. Blocking was done in 2% BSA, followed by incubation with primary and secondary antibodies. Images were taken in a CQ1 high throughput imaging system and analyzed using cell pathfinder software.

### Aβ estimation in plasma

After the end of the experiment, blood was collected from the retinal orbital plexus of mice in EDTA-coated vials. Samples were centrifuged, and plasma was separated. The levels of Aβ in the plasma were measured by using the protein-specific ELISA kit.

### Statistical analysis

Statistical analyses were performed using GraphPad Prism 8 software. All *in-vitro* experiments were performed thrice. The response of independent mice from the group was noted for animal experiments and the mean was calculated. The data presented here represents the mean ± SD. Statistical significance of the experiment was calculated using one-way ANOVA analysis and Bonferroni method was used as a *post-hoc* test. p value less than 0.05 was taken as significant.

## Results

### IBC induced autophagy and inhibited NLRP3 inflammasome in primary astrocytes

AMPK is a primary regulator of autophagy and NLRP3 inflammasome-mediated inflammation, and both mechanisms are dysregulated in AD. With this information, we identified IBC (purity-98.8%; Supplementary Figure S1) as an inducer of AMPK-mediated autophagy and an inhibitor of NLRP3 inflammasome. To prove the effect of IBC on these processes, we used mouse primary astrocytes for all the *in vitro* experiments. The purity of the culture was tested by using markers of astrocytes (glial fibrillary acidic protein; GFAP), neurons (neuronal nuclear protein; Neu N) and microglial cells (ionized calcium-binding adapter molecule 1; IBA1) (Supplementary Figures S2A, B). To analyze the effect of IBC on AMPK phosphorylation at Thr 172, the cells were treated for 24 h with IBC. The western blot analysis revealed a concentration-dependent increase in the expression of AMPK (Thr 172) (p < 0.0001 at 5, 10 and 20 μM; Figure 1B). We further found that the cells treated with IBC showed a significant increase in the levels of autophagy marker LC3B-II (Light chain 3) (p < 0.0001 at 5, 10 and 20 μM; Figure 1C). The induction of autophagy by IBC was also supported by reducing levels of another autophagy marker, SQSTM1 (sequestosome 1), with the increase of IBC concentration (p < 0.0001 at 10 and 20 μM; Figure 1C).

After confirming the induction of autophagy by IBC, we proceeded to know whether the ability of IBC to induce AMPK-mediated autophagy can lead to the inhibition of NLRP3 inflammasome. We found through ELISA that the primary astrocytes primed with media from LPS-stimulated microglial N9 cells, when activated with LPS (lipopolysaccharide) and ATP (5′ adenosine triphosphate), led to the release of IL-1β and IL-18. The release of both IL-1β and IL-18 was significantly inhibited with IC50 values 2.95 µM and 2.75 µM, respectively, when the cells were treated with IBC for 1.5 h, which clearly indicated the inhibition of NLRP3 inflammasome (Figures 1D, E). MCC950 (100 nM) was used as a standard inhibitor of NLRP3 inflammasome in the experiments. We further confirmed the inhibition of NLRP3 inflammasome by measuring the cleaved and active forms of IL-1β and CASP1 from the supernatant of cells treated with IBC by using western blotting. The data clearly indicated the reduced release of IL-1β and CASP1 (p < 0.0001 at 5 and 10 μM) from the cells and, thus, the inhibition of NLRP3 inflammasome, though there was no change in the expression of NLRP3 protein (Figure 1F). Further, to check the effect of IBC on NLRP3 inflammasome complex formation, we analyzed the levels of ASC protein oligomers. Results showed that IBC-treated cells inhibited ASC monomer, dimer, and oligomer formation (Supplementary Figures S2C, D). Further, the IBC treatment at 5 μM and 10 µM concentrations showed a highly significant (p < 0.0001) reduction of speck formation in cells, in comparison to the cells induced with ATP, indicating reduced oligomerization (Supplementary Figures S2E, F).

### IBC-induced autophagy by CAMKK2-mediated activation of AMPK pathway

**FIGURE 2:**
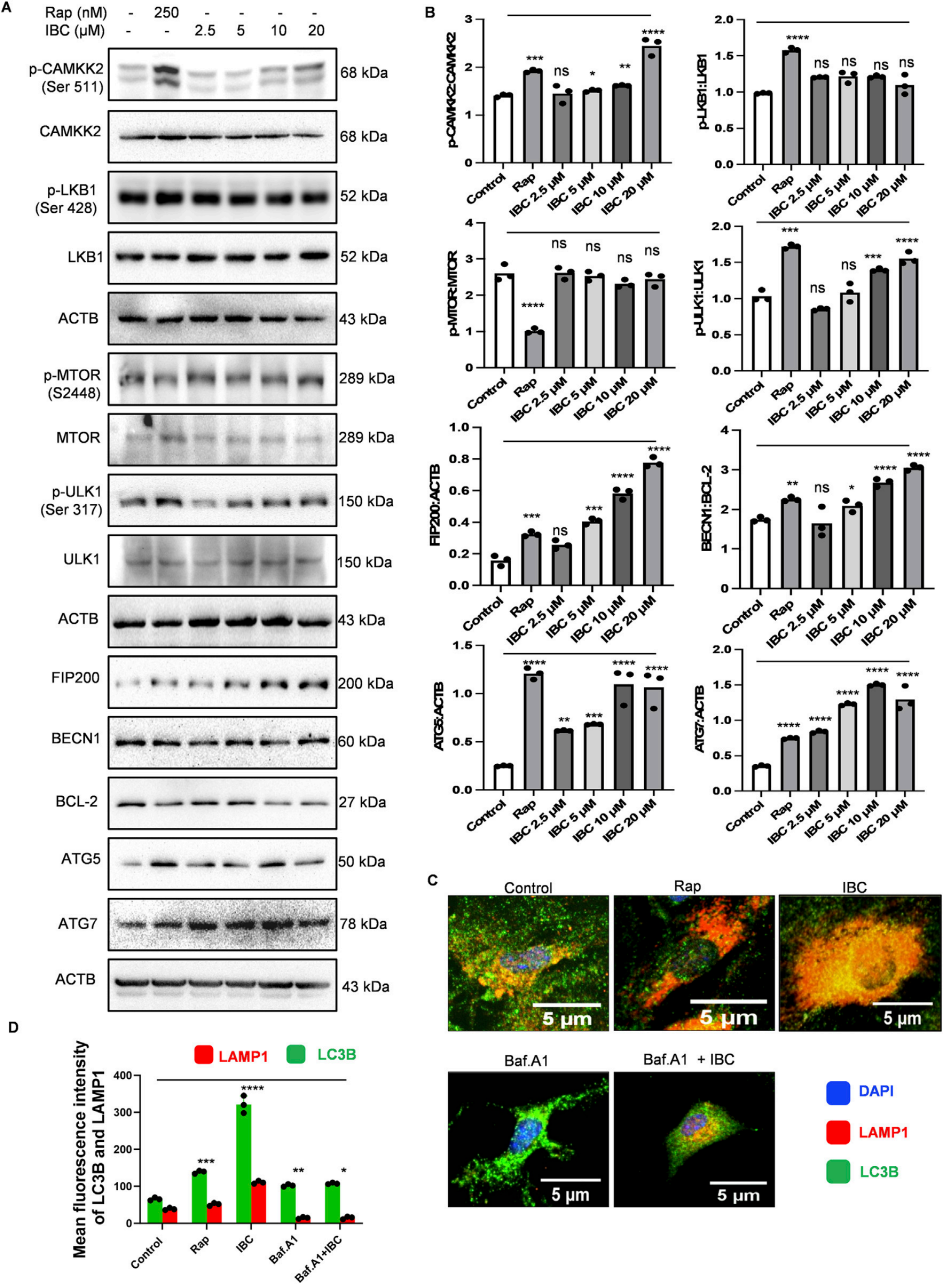
IBC upregulates autophagy induction in primary astrocytes. Primary astrocytes were treated with IBC or Rap for 24 h. **(A)** Immunoblots representing the effect of IBC on autophagy pathway proteins. **(B)** Densitometry analysis of immunoblots given in Figure 2A; p-CAMKK2: CAMKK2, p-LKB1: LKB1, p-MTOR: MTOR, p-ULK1: ULK1, FIP200: ACTB, BECN1: BCL2, ATG5: ACTB and ATG7: ACTB. Densitometry analyses were done using ImageJ software. **(C)** Representative confocal images depicting the effect of IBC (10 μM) on LAMP1 (red) and LC3B (green) co-localization in the presence and absence of autophagy inhibitor bafilomycin A_1_ (Baf.A1); nuclei stained by DAPI (blue). Full images of Figure 2C are given in Supplementary Figure S3B. **(D)** Graph representing mean fluorescence intensity of Figure 2C images. Images were acquired and analyzed by CQ1 high throughput imaging system. Scale bars were drawn by ImageJ software. Statistical significance was measured by one-way ANOVA and Bonferroni test as *post-hoc* (n = 3). p values ****p < 0.0001, ***p < 0.001, **p < 0.01, *p < 0.05. Rap, Rapamycin; IBC, Isobavachalcone; ACTB, Actin B; CAMKK2, calcium/calmodulin-dependent protein kinase kinase 2; LKB1, liver kinase B1; MTOR, mechanistic target of rapamycin kinase; ULK-1, unc-51-like kinase 1; FIP200, Focal adhesion kinase family Interacting Protein of 200 kD; BCL2, B-cell lymphoma; BECN1, beclin 1; ATG, autophagy related; LAMP-1, lysosomal-associated membrane protein 1; LC3, light chain 3.

Before moving further, we wanted to know if IBC has any toxicity in primary astrocytes. Therefore, we assessed the toxicity using two different assays, MTT and SRB. In both assays, IBC showed no toxicity in astrocytes even at 100 μM concentration (Supplementary Figure S3A). The activation of AMPK is regulated by two main kinases, LKB1 (liver kinase B1) and CAMKK2 (calcium/calmodulin-dependent protein kinase kinase 2). To ascertain the cause of AMPK activation, we checked the phosphorylation and activation of these kinases after treating primary astrocytes with IBC for 24 h. Interestingly, there was no change in the levels of pLKB1 (Ser 428), whereas pCAMKK2 (Ser 511) was found to be significantly upregulated after concentration-dependent treatment with IBC (p < 0.05 at 5 μM, 0.01 at 10 μM and 0.0001 at 20 μM; Figures 2A, B). However, we found no change in MTOR (mechanistic target of rapamycin) levels. ULK1 (unc-51-like kinase 1), which is a direct target of AMPK, was found to be phosphorylated at Ser 317, marking its activation; which was also confirmed indirectly through increased phosphorylation of FIP200, another protein present in the ULK1 complex (Figures 2A, B). Further, the downstream protein BECN1 (beclin 1, autophagy related) did not show any change in the expression. However, BCL2 (B-cell lymphoma), which is known to interact with BECN1 and downregulate autophagy, showed a marked decline, thereby tilting the ratio in favor of BECN1 activation (p < 0.05 at 5 μM, and 0.0001 at 10 and 20 μM; Figures 2A, B). The level of proteins ATG5 (autophagy related 5) and ATG7 (autophagy related 7), involved in the maturation of the autophagosome, was also found to be upregulated, thus confirming the induction of autophagy by IBC in primary astrocytes (Figures 2A, B). To confirm the fusion of autophagosome with the lysosome, we did a confocal microscopy analysis for the co-localization of LC3B and LAMP1 (lysosomal-associated membrane protein 1), membrane marker proteins for autophagosome and lysosome, respectively. The data showed the proximity of LC3B and LAMP1 after the treatment of primary astrocytes with IBC (p < 0.0001 at 10 μM; Figures 2C, D; Supplementary Figure S3B). In all these experiments, rapamycin (Rap) at a concentration of 250 nM was used as a standard drug for the induction of autophagy.

### IBC inhibited NLRP3 inflammasome through autophagy induction in primary astrocytes

**FIGURE 3:**
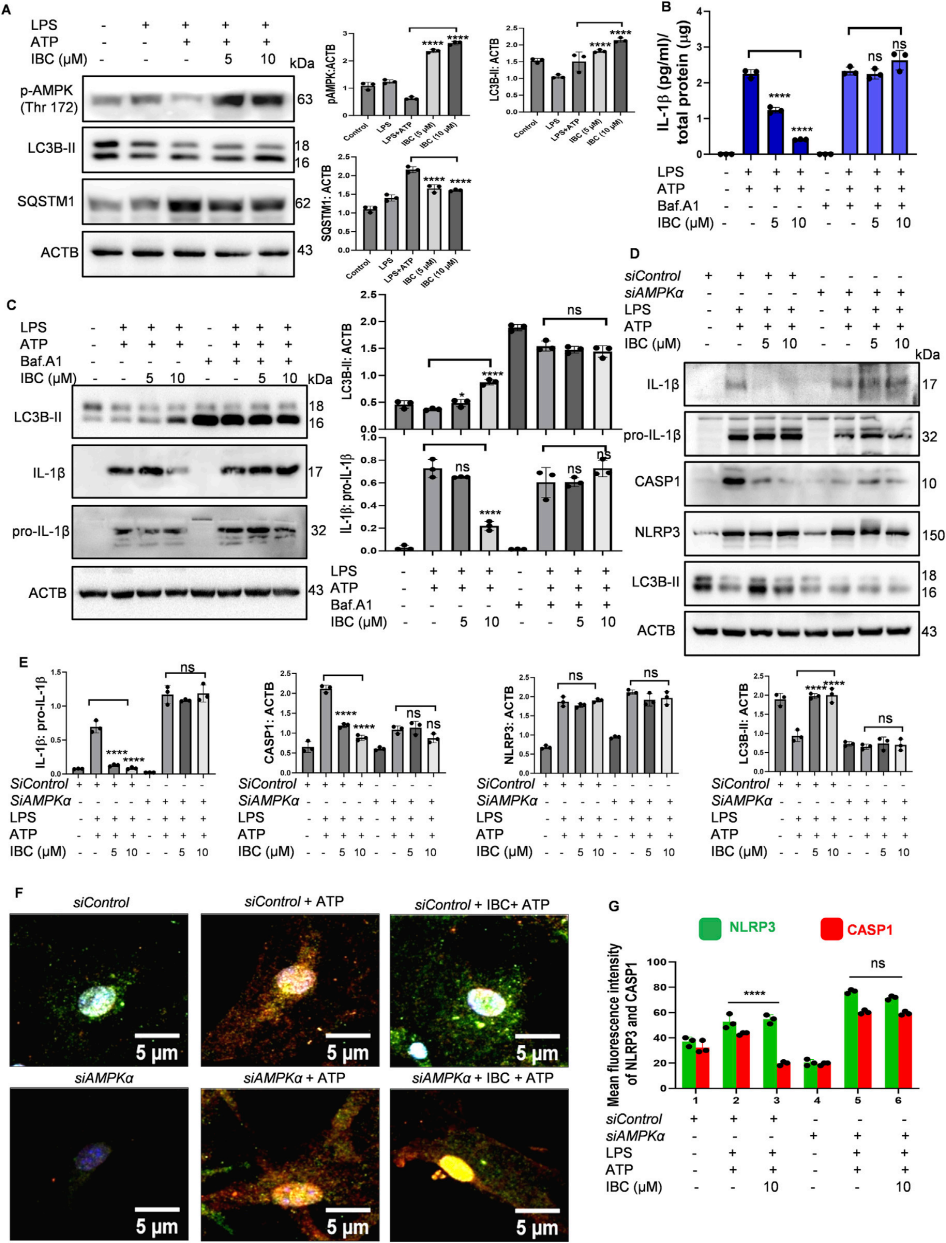
IBC inhibits NLRP3 inflammasome through AMPK-mediated autophagy. Primary astrocytes were treated for NLRP3 activation as described in Figure 1, and the effect on autophagy pathway proteins was analyzed. **(A)** Immunoblots and densitometry analysis of autophagy marker proteins. Primary astrocytes before treatment for NLRP3 inflammasome activation, were pre-treated with 20 nM bafilomycin A_1_ (Baf.A1) for 1 h. **(B)** Levels of IL-1β measured through ELISA. **(C)** Levels of LC3B-II and IL-1β analyzed through immunoblotting. AMPK protein level was knocked down from primary astrocytes using s*iAMPK*. **(D)** Immunoblots to check the levels of proteins after reduced AMPK expression. **(E)** Densitometry analyses of Figure 3D immunoblots; IL-1β: pro-IL-1β CASP1: ACTB, NLRP3: ACTB and LC3B-II: ACTB. **(F)** Representative images demonstrating the effect of IBC (10 μM) on NLRP3 (green) and CASP1 (red) co-localization; nuclei stained by DAPI (blue). **(G)** Graph representing mean fluorescence intensity of images shown in Figure 3F. Full images of Figure 3F are given in Supplementary Figure S4A. Images were taken and analyzed in CQ1 high throughput imaging microscope. Densitometry analyses and scale bars were inserted using ImageJ software. Data presented was statistically analyzed through a one-way ANOVA test followed by the Bonferroni test as *post hoc* (n = 3). p values ****p < 0.0001, ***p < 0.001, **p < 0.01, *p < 0.05. IBC, Isobavachalcone; ACTB, Actin B; AMPK, 5′ adenine monophosphate-activated protein kinase; LC3, light chain 3; SQSTM1, Sequestosome 1; LPS, lipopolysaccharide; ATP, 5′ adenosine triphosphate; CASP1, caspase1; IL-1β, interleukin-1β; NLRP3, NOD-like receptor family pyrin domain containing 3.

After confirming the induction of autophagy by IBC, we wanted to know if it plays any role in inhibiting NLRP3 inflammasome in primary astrocytes. In our earlier assays, autophagy was checked after 24 h of treatment, and the inhibition of NLRP3 inflammasome was analyzed after 90 min of treatment with IBC. Therefore, we first checked whether IBC could induce autophagy under the conditions used for the NLRP3 inhibition assay. Interestingly, cells treated with IBC in the presence of LPS and ATP showed significantly enhanced phosphorylation of AMPK at Thr 172, along with that a marked increase in the level of autophagy marker LC3B-II was observed (p < 0.0001 at 5 and10 μM; Figure 3A). Further, the additional autophagy marker SQSTM1 levels also showed a significant decline after treatment with IBC (p < 0.0001 at 5 and10 μM; Figure 3A). To confirm the involvement of autophagy in NLRP3 inhibition by IBC, we inhibited the autophagy by end-stage autophagy inhibitor bafilomycin A_1_ and checked its effect on NLRP3 inflammasome mediated release of IL-1β. We first checked the inhibition of autophagy by analyzing the expression of LC3B-II, which showed an accumulation of the protein in the presence of bafilomycin A_1_, thus confirming the inhibition of autophagy. The inhibition of autophagy completely reversed the inhibitory effect of IBC on the release of IL-1β, which was almost equal to the levels in the cells treated with LPS + ATP as confirmed by both ELISA and western blotting (Figures 3B, C). These results clearly indicated the autophagic regulation of IBC-mediated inhibition of NLRP3 inflammasome.

Our earlier data showed that AMPK plays an essential role in autophagy induced by IBC. Therefore, we wanted to know if the knockdown of AMPK by using siRNA can affect the inhibition of NLRP3 inflammasome by IBC. We found that under the conditions of NLRP3 inhibition assay, the *siRNA*-mediated inhibition of AMPK completely reversed the inhibitory effect of IBC on two important proteins (IL-1b and CASP1) of the NLRP3 inflammasome complex. The release of both these proteins after treatment with IBC in the presence of *siAMPK* was almost similar to that of astrocytes treated with LPS + ATP (Figures 3D, E). Interestingly, there was no change in the expression of NLRP3 protein in the presence or absence of *siAMPK,* whereas its negative effect on autophagy marker LC3B-II was clearly visible (Figures 3D, E). The effect of *siAMPK* on the assembly proteins involved in NLRP3 complex formation also showed a similar trend. Treating astrocytes with IBC reduced the co-localization of CASP1 and NLRP3 compared to those treated with LPS + ATP (p < 0.0001 at 10 μM; Figures 3F, G; Supplementary Figure S4A). However, in the presence of *siAMPK,* the cells treated with IBC did not affect the co-localization of CASP1 and NLRP3 as observed through confocal microscopy (Figures 3F, G; Supplementary Figure S4A). Western blotting and confocal microscopy confirmed the knockdown effect of *siAMPK* on the AMPK (Thr 172) in all the experiments (Supplementary Figures S4B, C, D).

### Autophagy induced by IBC promotes the clearance of Aβ in primary astrocytes

**FIGURE 4:**
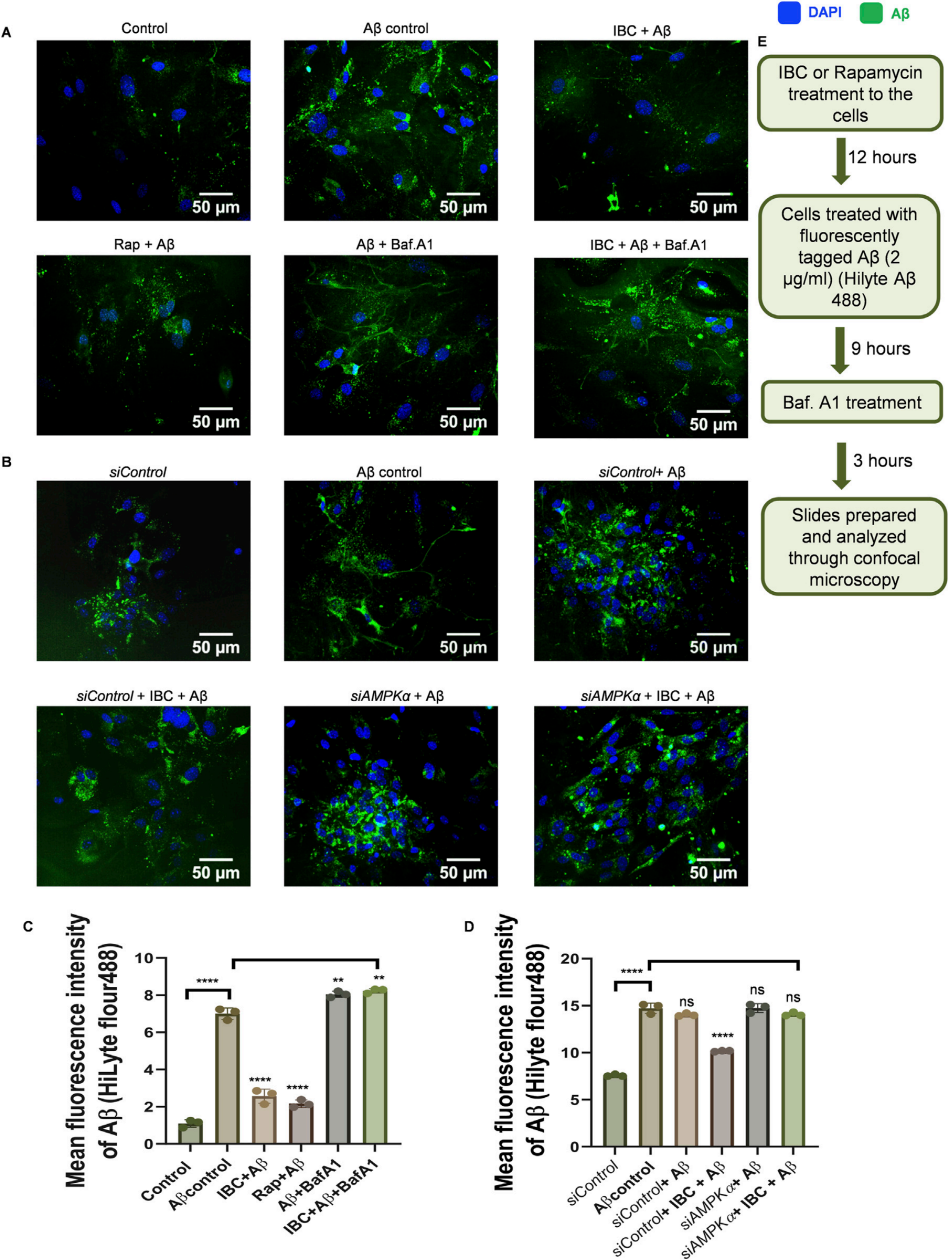
IBC promotes clearance of Aβ through AMPK-regulated autophagy. Primary astrocytes were treated with 2 μg/ml fluorescently tagged (HiLyte 488) Aβ peptide for 12 h after IBC (10 μM) treatment to cells, in the presence or absence of bafilomycin A_1_ (Baf.A1). **(A)** Images representing the effect of IBC on intracellular Aβ (green) in primary astrocytes. After the transfection of cells to reduce AMPK expression, primary astrocytes were treated similarly as in Figure 4A. **(B)** Representative images of IBC effect on Aβ (green) in transfected primary astrocytes); nuclei were stained by DAPI (blue). **(C)** Mean fluorescence intensities of images shown in Figure 4A. **(D)** Mean fluorescence intensities of images are shown in Figure 4B. Images were acquired and analyzed by CQ1 cell pathfinder software. **(E)** Experimental design to assess Aβ clearance given in Figure 4A. Scale bars were drawn using ImageJ software. Data was analyzed through one-way ANOVA analysis and Bonferroni test as *post hoc* (n = 3).p values ****p < 0.0001. Rap, Rapamycin; IBC, Isobavachalcone; Baf.A1, Bafilomycin A1; DAPI, 4,6-diaminido-2-phenylindole; Aβ, amyloid-beta.

After confirming the dual pharmacological activity of IBC, we further wanted to determine whether the autophagic activity of IBC can help glial cells to clear Aβ. Therefore, we treated the astrocytes with IBC for 24 h and added Aβ_42_-HiLyte fluor488 to the culture 12 h before the termination of the experiment. The confocal image analysis revealed that the cells with IBC showed significantly low fluorescence compared to the cells with Aβ alone, indicating reduced intracellular levels of Aβ in the IBC-treated cells (p < 0.0001). However, when we inhibited the autophagy with bafilomycin A_1_, the levels of Aβ were found to be increased in the treated cells, which confirmed the involvement of autophagy in the clearance of Aβ by IBC (Figures 4A, C). These results were further consolidated when we inhibited autophagy at an early stage by using *siRNA* against AMPK. The cells treated with IBC displayed a marked decline in intracellular levels of Aβ compared to Aβ control. The cells that were treated with *siAMPK* showed a complete reversal of the IBC effect on the clearance of Aβ (Figures 4B, D). The treatment plan for these experiments is given in Figure 4E.

### IBC crossed the blood-brain barrier and improved the cognitive deficits in 5x-FAD mice

**FIGURE 5:**
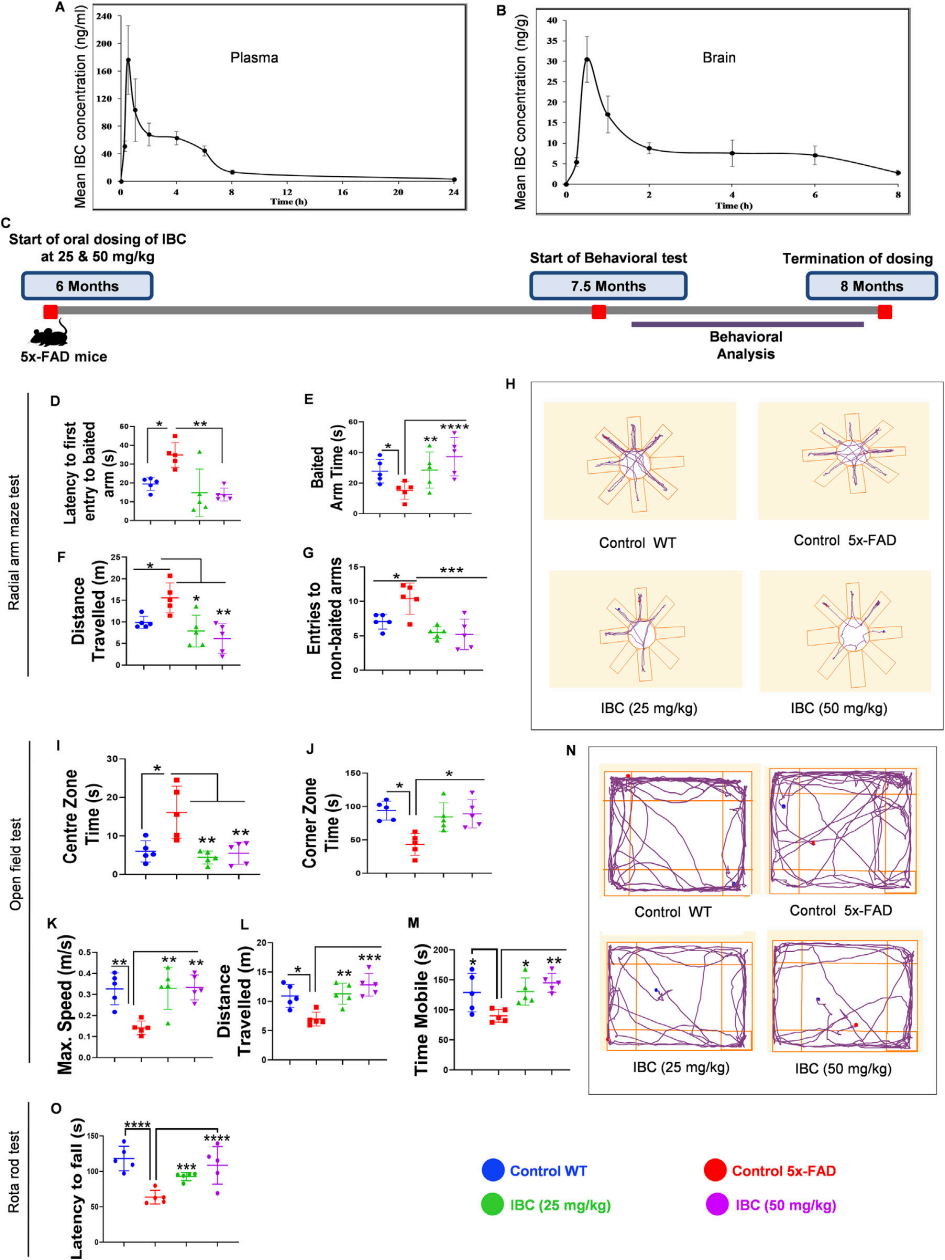
IBC mitigates the cognitive impairments in transgenic mice model of AD. Pharmacokinetic analysis of C57BL/6J mice after 30 mg/kg oral dose of IBC (n = 5). **(A)** Mean plasma concentration vs. time profile of IBC. **(B)** Mean brain concentration vs. time profile of IBC. Main PK parameters are presented in Tables 1 and 2. IBC improves the pathophysiology of 5x-FAD mice. Post-dosing of 5x-FAD mice with IBC at 25 mg/kg and 50 mg/kg concentration, behavioral studies were performed to check the effect on cognitive decline in AD mice. Untreated 5x-FAD control mice were compared with age-matched wild-type C57BL/6J mice. Post 5 days training of mice to locate bait in a radial arm maze with visual clues, the IBC effect on mice’s spatial memory was evaluated (n = 5). **(C)** Study timeline of IBC treatment in 5x-FAD mice (n = 5) (detailed study design is given in Supplementary Figure S5). Graph representing **(D)** Latency to first entry to baited arm, **(E)** Baited arm time, **(F)** Distance traveled and **(G)** Entries to non-baited arm in radial arm maze test. **(H)** Representative track plots of mice in radial arm maze. To investigate the effect of IBC on the behavioral and locomotor activity of mice, an open-field test was carried out. Graphs displaying **(I)** Centre zone time, **(J)** Corner zone time, **(K)** Maximum speed, **(L)** Distance traveled, and **(M)** Time mobile of mice in the open field. **(N)** Representative track plots of mice in the open field. After training mice to run on rotarod, analysis was done to check their motor coordination and balance. **(O)** Graph depicting latency to fall of mice from rotarod apparatus. The activity of mice in the radial arm maze and open field test was recorded by an automated camera and analyzed through AnyMaze software. The statistical significance of data was measured by one-way ANOVA, followed by the Bonferroni test as *post-hoc.* p values ****p < 0.0001, ***p < 0.001, **p < 0.01, *p < 0.05. IBC, Isobavachalcone; WT, wild type.

Based on *in vitro* data, we decided to investigate the effect of IBC on the pathology of AD in the transgenic mouse model (5x-FAD). Before going for a long-term study to analyze the effect of IBC on AD pathology, we first confirmed the penetration of IBC into the brain after a single dose (p.o.). Therefore, we compared the plasma and brain pharmacokinetic (PK) profile of IBC after a single dose of 30 mg/kg (p.o). The blood samples collected at different time points through 24 h showed plasma C_max_ value of 208 ± 46 ng/ml (Figure 5A; Table 1). The brain PK studies revealed that IBC crossed the blood-brain barrier with low penetration and showed a C_max_ value of 34 ± 04 ng/g of brain tissue (Figure 5B; Table 2). We determined the brain-to-plasma ratio of 0.12–0.16. To observe the effect of IBC on AD pathology, we decided to treat the 6 months old 5x-FAD mice at two different doses that are 25 mg/kg and 50 mg/kg, for 2 months (Experiment timeline is given in Figure 5C and detailed plan is illustrated in Supplementary Figure S5).

**TABLE 1:** The pharmacokinetic parameters of IBC in blood plasma after oral administration at 30 mg/kg in C57BL/6J mice.

Parameters	IBC (30 mg/kg)
C_max_ (ng/mL)	208 ± 46
T_max_ (h)	0.6 ± 0.1
T_1/2_ (h)	5.6 ± 1.5
AUC_0-t_ (ng.h/mL)	548 ± 59
AUC_0-∞_ (ng.h/mL)	637 ± 74
V_d_ (L/Kg)	355 ± 81
Cl (L/h/Kg)	50 ± 7

**TABLE 2:** Main pharmacokinetic parameters of IBC in brain after 30 mg/kg oral administration in C57BL/6J mice (n = 5).

Parameters	IBC (30 mg/kg)
C_max_ (ng/g)	34 ± 4
T_max_ (h)	0.6 ± 0.1
T_1/2_ (h)	1.8 ± 0.4
AUC_0-t_ (ng.h/g)	72 ± 7
AUC_0-∞_ (ng.h/g)	79 ± 7

After 6 weeks of dosing, the animals were trained for 5 days each for the radial arm maze and rotarod tests. We generated the baseline behavior data by comparing the age-matched wild-type C57BL/6J mice group with the 5x-FAD control group and found a significant deviation in all the parameters tested during behavior analysis. In a radial arm maze test, the animals treated with IBC at both the doses 25 and 50 mg/kg took significantly less time (16.58 and 14.36 s, respectively) to enter the baited arm in comparison to the control group, which took an average of 32.3 s (p < 0.01; Figure 5D). Furthermore, the animals treated with IBC spent a significantly longer time in the baited arm than the control group (p < 0.01 at 25 mg/kg and <0.0001 at 50 mg/kg; Figure 5E). The treated animals also traveled significantly less distance to find the baited arm and showed a smaller number of entries into the non-baited arm (Figures 5F, G). These data clearly indicated improvement in spatial memory of 5x-FAD mice treated with IBC. We also tested the exploratory behavior of mice. The 5x-FAD mice displayed a significant reduction in their desire to explore the surrounding environment, which represents the behavior associated with AD. However, the animals treated with IBC showed improved exploratory behavior, which was evident from less time spent in the center, more time spent in the corners, and being in the mobile state for a longer time at higher speed along with greater distance traveled as compared to the 5x-FAD control group (p < 0.05; Figures 5I–M). The track plots of the mice in the radial arm maze test and open field test, which were drawn using AnyMaze software, are shown in Figures 5H, N, respectively. The treatment of 5x-FAD mice with IBC also improved their neuro-muscular coordination and stamina as evaluated by the rotarod test. The animals treated with IBC were able to stay on moving rods for a significantly longer time (92 s at 25 mg/kg and 118 s at 50 mg/kg) in comparison to the 5x-FAD control group, which could stay on rods for about 63 s only (p < 0.001 at 25 mg/kg and <0.0001 at 50 mg/kg; Figure 5O).

### Improvement in the memory behavior in 5x-FAD mice is associated with IBC-mediated clearance of Aβ_42_ and reduction of neuroinflammation

**FIGURE 6:**
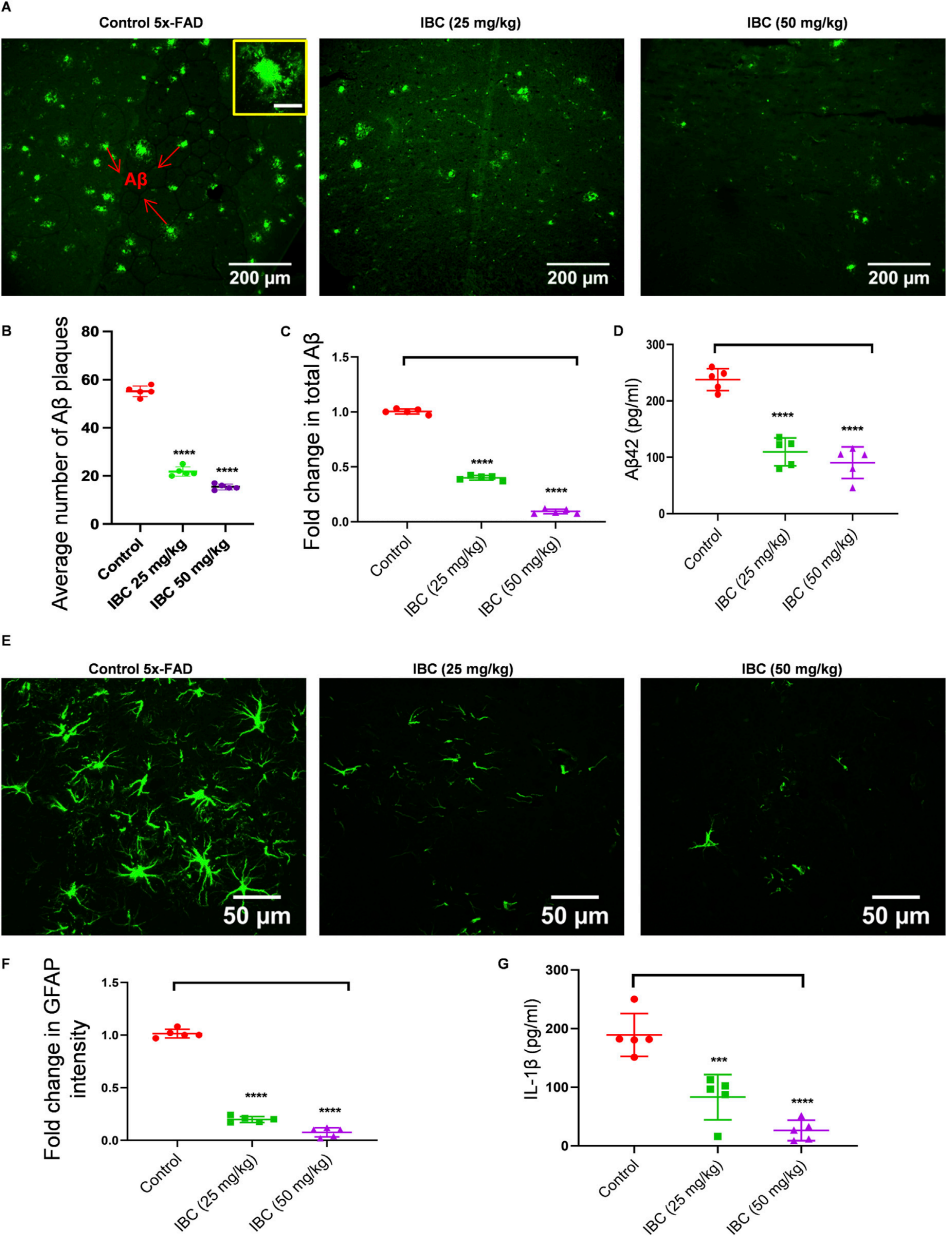
IBC alleviates Aβ deposition and neuroinflammation in 5x-FAD mice. Post-dosing, mice hippocampus tissue was analyzed for Aβ deposition using an IHC assay (n = 5). **(A)** Representative images of mice hippocampus tissue fluorescently stained with anti-Aβ antibody (green). The inset shows a magnified image of a single Aβ plaque with 20 μm scale bar. Graph showing **(B)** average number of Aβ plaques and **(C)** fold changes in total Aβ intensity, amongst groups. **(D)** Levels of Aβ in mice blood plasma. Reactive astrocytes were seen in hippocampus. **(E)** Representative images of mice hippocampus tissue fluorescently stained with anti-GFAP antibody (green). **(F)** Graph depicting fold changes in GFAP intensity. **(G)** Levels of IL-1β measured in mice cortex by ELISA. Fluorescence images merged with brightfield images of Figures 6A,E are given in Supplementary Figures S6A, C, respectively. Higher magnification images showing deposition of Aβ at CA region is given in Supplementary Figure S6B. Images were taken and analyzed using cell pathfinder software of CQ1 imaging system. Scale bars were inserted by ImageJ software. Statistical analyses were done using one-way ANOVA and Bonferroni test for multiple comparisons. p values ****p < 0.0001, ***p < 0.001. IBC, Isobavachalcone; Aβ, amyloid-beta; IL-1β, interleukin-1β; GFAP: glial fibrillary acidic protein.

The improved memory behavior of 5x-FAD mice after 2 months of treatment with IBC led us to explore the molecular changes in the brains of these mice. Therefore, we analyzed the effect of IBC on the deposition of Aβ, which is a key pathological feature of AD responsible for cognitive impairment. We evaluated the Aβ levels in the hippocampi of the 5x-FAD mice by immunohistochemical (IHC) analysis. We observed a huge load of Aβ plaques (Average number of plaques = 55) in the 5x-FAD control mice group. The counting of Aβ plaques, using the cell pathfinder software, revealed that the average number of plaques was reduced to 21 and 15 in the mice that were treated with IBC at 25 and 50 mg/kg, respectively (p < 0.0001; Figures 6A, B; Supplementary Figures S6A, B). This was further validated by the fluorescence analysis of samples, which displayed a highly significant decline in the treated groups (Figures 6A, C). Further, the analysis through ELISA also exhibited reduced levels of Aβ_42_ in the blood plasma of mice treated with IBC compared to the untreated control group, indicating the decline in the Aβ load in these mice (p < 0.0001; Figure 6D).

Deposition of Aβ_42_ is strongly linked with increased neuroinflammation, which is reflected in the form of reactive astrogliosis and activation of NLRP3 inflammasome. Thus, we intended to know if the IBC meditated decline in Aβ_42_ has reduced brain inflammation. The IHC analysis of the hippocampi for GFAP showed a large number of astrocytes expressing elevated levels of GFAP in the control group. However, in the mice treated with 25 and 50 mg/kg of IBC, the expression of GFAP was significantly reduced (p < 0.0001; Figures 6E, F; Supplementary Figure S6C). Also, Aβ, being one of the damage-associated molecular patterns (DAMP) known to activate NLRP3 inflammasome, we checked if the dwindling load of Aβ after treatment with IBC could reduce the activation of NLRP3 inflammasome. For that, we analyzed the levels of IL-1β in the cortices in the brains of these mice using ELISA. The data indicated a highly significant decline in the levels of IL-1β in the 5x-FAD mice treated with IBC at both doses 25 and 50 mg/kg, compared to the control group (p < 0.001 at 25 mg/kg and <0.0001 at 50 mg/kg; Figure 6G).

### IBC induced autophagy and inhibited NLRP3 inflammasome in the hippocampus of 5x-FAD mice

**FIGURE 7:**
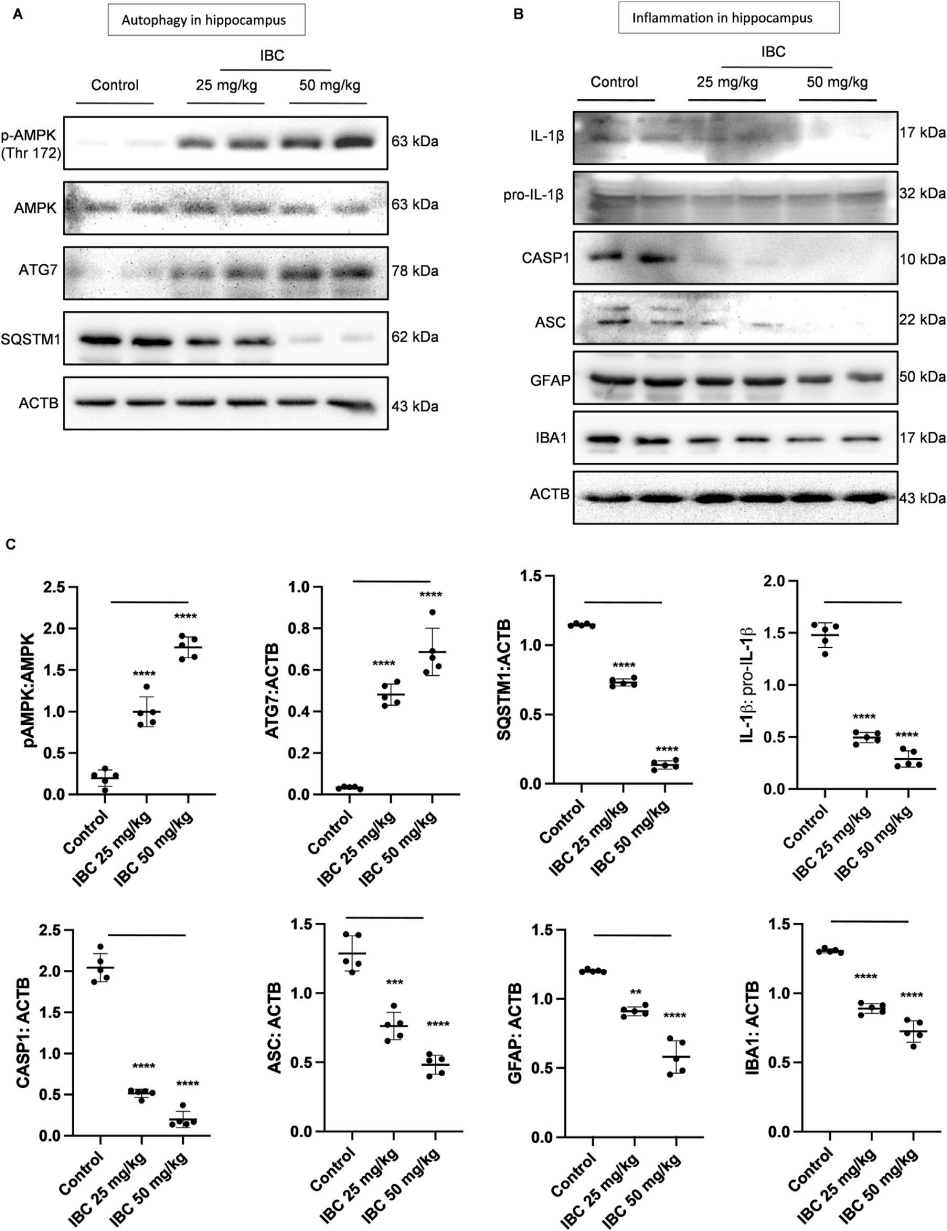
IBC induces autophagy and ameliorates neuroinflammation in 5x-FAD mice hippocampus (n = 5). **(A)** Immunoblots of autophagy pathway proteins. **(B)** Immunoblots of NLRP3 inflammasome and neuroinflammation marker proteins. **(C)** Densitometry of Figures 7A, B, analyzed by ImageJ software. Statistical analyses were performed through one one-way ANOVA test followed by the Bonferroni test as *post-hoc*. p values ****p < 0.0001, ***p < 0.001, **p < 0.01. IBC, Isobavachalcone; AMPK, 5′ adenine monophosphate-activated protein kinase; SQSTM1, Sequestosome 1; ATG, autophagy related; ACTB, Actin B; CASP1, caspase1; ASC, Apoptosis-associated Speck-like protein containing a Caspase recruitment domain; AMPK, 5′ adenine monophosphate-activated protein kinase; LC3, light chain 3; SQSTM1, Sequestosome 1; IL-1β, interleukin-1β; GFAP: glial fibrillary acidic protein; IBA1, ionized calcium-binding adapter molecule 1.

After confirming the clearance of Aβ deposits and alleviation of inflammation from the brain of 5x-FAD mice, we analyzed the expression of some of the proteins that are directly involved in the autophagic pathway. The western blot analysis clearly showed increased expression of pAMPK and ATG7, whereas the expression of SQSTM1 was significantly decreased in the mice treated with IBC at 25 and 50 mg/kg (p < 0.0001; Figure 7A). Further, the animals treated with IBC also expressed reduced levels of NLRP3 inflammasome proteins. The western blot analysis showed significant abrogation of CASP1 (p < 0.0001) and ASC (p < 0.001 at 25 mg/kg and <0.0001 at 50 mg/kg) in mice treated with IBC. This was further reflected in the reduced cleavage of IL-1β in these mice (p < 0.0001; Figure 7B). The treatment of mice with IBC also inhibited the level of the reactive phenotype of astrocytes and microglial cells, evidenced by decreased expression of GFAP (p < 0.01 at 25 mg/kg and <0.0001 at 50 mg/kg) and IBA1 (p < 0.0001) (Figure 7B).

## Discussion

The complex pathology of AD has led to the failure of multiple drugs in clinical trials. The failure rate (99.6%) of anti-AD drugs in clinical trials is the highest among all the diseases (Cummings et al., 2017; Cummings et al., 2014). Though, three anti- Aβ biologicals, viz. aducanumab, lecanemab-irmb, and donanemab have recently been approved by the US FDA for treating AD (Cummings, 2023; Sevigny et al., 2016; van Dyck et al., 2023). However, the efficacy of these biologicals to provide long-term clinical benefits to AD patients will be decided in the future. The clinical trial data analysis shows that most drugs that failed against AD were based on a single target. This study was designed to employ the idea that hitting more than one AD target, that are, Aβ and NLRP3 inflammasome-mediated neuroinflammation, could effectively ameliorate AD pathology. To implicate this concept, we chose an experimental compound, IBC. IBC induces AMPK-mediated autophagy in primary astrocytes. The autophagy induction by IBC lead to the inhibition of NLRP3 inflammasome. Additionally, IBC also induces autophagy-dependent Aβ clearance from primary astrocytes. We found that IBC caused the induction of CAMKK2 to activate AMPK and its downstream targets, which ultimately led to the completion of autophagy. Before assessing the pharmacological effect of IBC, we did preliminary cytotoxicity studies, which revealed that the compound is non-toxic to primary astrocytes up to 100 µM concentration. We further profiled the concentration-dependent effect of IBC on NLRP3 inflammasome inhibition and autophagy induction. Since IBC showed a significant effect at 10 μM, this concentration was used for imaging assays *in vitro* (Sofroniew and Vinters, 2010; Garwood et al., 2017; Gonzalez-Reyes et al., 2017). Autophagy is the major cellular pathway that is known to modulate the activation of the NLPR3 inflammasome (Houtman et al., 2019; Shi et al., 2012). It clears the pro-form of proteins from the cytoplasm and manages the clearance of NLRP3 protein ligands like ROS-producing dysfunctional mitochondria (Harris et al., 2011; Rodgers et al., 2014). In the AD brain, due to the continuous death of cells, the release of free ATP becomes a source for activation of NLRP3 inflammasome (Guo et al., 2020; Huang et al., 2021). Additionally, Aβ is another DAMP responsible for chronic NLRP3 inflammasome activity and release of IL-1β in AD (Luciunaite et al., 2020). IBC, through autophagy induction, limited the ATP-mediated NLRP3 inflammasome activation by inhibiting ASC oligomerization and speck formation. The involvement of autophagy in anti-NLRP3 inflammasome was confirmed when we inhibited the autophagy at two different steps. The inhibition of autophagy at the initial stage by using *siAMPK* and at the final stage by bafilomycin A_1_ led to a reversal of the anti-NLRP3 inflammasome activity of IBC. Further, IBC displayed the auxiliary advantage of clearing Aβ deposits in the primary astrocytes through autophagy induction. Thus, IBC acted on two of the essential DAMPs in the brain responsible for NLRP3 inflammasome-mediated neuroinflammation.

After confirming the *in vitro* activity of IBC against Aβ clearance and NLRP3 inflammasome, the two important targets in AD, we validated its efficacy in the 5x-FAD transgenic mouse model of the disease. Before that, we confirmed the IBC’s penetration into the brain through PK studies in C57BL/6J mice, a genetic background for transgenic 5x-FAD mice. The single 30 mg/kg dose of IBC showed a low brain availability. Hence, we planned to treat the 5x-FAD mice at two different doses of IBC (25 and 50 mg/kg, p.o.) for 2 months, where the higher dose was chosen to increase the brain penetration of IBC. We found that even though the single dose of IBC had shown low brain availability, the repeated doses of IBC displayed significant improvement in AD pathology in 5x-FAD mice, even at the lower dose of 25 mg/kg. AD pathology progresses with time, and some of the latest antibodies-based clinical trials targeting Aβ have indicated that starting treatment at an early stage of AD may be helpful in better clinical outcomes (Habashi et al., 2022; Yiannopoulou et al., 2019). Therefore, we began the treatment of 5x-FAD mice at 6 months of age, which is an early stage of the disease (Forner et al., 2021).

The analysis of all the parameters related to cognitive behavior clearly indicated the impairment in 5x-FAD control mice compared to wild-type C57BL6/J mice. However, the IBC-treated groups showed significant improvement in spatial memory. Along with spatial memory, the decline in other behavior parameters is a common feature of AD (Albert, 1996; Pottorf et al., 2022; Webster et al., 2014). Therefore, we also analyzed the exploratory behavior of mice after treatment with IBC. The 5x-FAD mice displayed deficits in the parameters related to exploratory behavior and reduced neuromuscular coordination, which indicated deterioration of brain function. The mice treated with IBC displayed a reversal of functional decline in the brain at both the doses used for treatment. Intriguingly, the mice treated with IBC performed better than the wild-type mice in most of the evaluated parameters. This observation could be attributed to improved autophagic homeostasis and reduced NLRP3-mediated chronic inflammation, which helped the treated mice perform better.

We further confirmed whether the improvement in behavior parameters is related to the effect of IBC on the clearance of Aβ and reduction of inflammation through autophagy, as observed in the *in vitro* studies. Several recent studies have strongly linked the decline of autophagy as one of the reasons for the deposition of Aβ in the AD brain (Lee et al., 2022; Wani et al., 2021; Luo et al., 2020). Moreover, a complex cascade of events follows the deposition of Aβ, which involves the release of inflammatory cytokines by microglia and astrocytes, leading to the death of neurons (Kitazawa et al., 2004; Singh, 2022; Wu et al., 2013). Therefore, we analyzed the hippocampi of mice treated with IBC for Aβ deposits. The data confirmed that autophagy induced by IBC effectively cleared Aβ_42_ plaques from the hippocampus at 25 and 50 mg/kg doses. Furthermore, the plasma levels of Aβ_42_ were also significantly reduced, thereby confirming the effect of IBC on its clearance from the brain.

The analysis of autophagic proteins like AMPK, ATG7, and SQSTM1, which we earlier observed in astrocytes, were also regulated in the hippocampi of treated mice. These data further emphasized the mechanism of action of IBC for clearance of Aβ from the brain. Apart from the deposition of Aβ, neuroinflammation is one of the critical hallmarks of AD. The release of pro-inflammatory cytokine IL-1β due to chronic activation of NLRP3 inflammasome by Aβ, TAU, and ATP creates an inflammatory microenvironment in the brain, contributing further to neurodegeneration (Ising et al., 2019; Akiyama et al., 2000; Heneka et al., 2018). In this study, IBC treatment mitigated the neuroinflammation in 5x-FAD mice, which was confirmed by reduced GFAP and IL- 1β expression in the hippocampi and cortex respectively. Furthermore, the expression levels of ASC and CASP1, the essential proteins of NLRP3 inflammasome complex, were significantly reduced in the mice treated with IBC, suggesting the involvement of autophagy in the inhibition of NLRP3 inflammasome in the hippocampus of mice treated with IBC.

Aβ deposits are one of the primary markers for AD neuropathology (DeTure and Dickson, 2019). So, we measured the effect size of IBC on the Aβ levels which was large (Cohen’s d > 0.8) (Sullivan and Feinn, 2012). For preclinical assessment of neuropathological outcomes in 5x-FAD mice, large effect size or normalization of neuroinflammation are suggested appropriate to be measured in smaller cohort (n < 10) (Faisal et al., 2023). This verifies that even with a small sample size, our data can be clinically significant for further evaluation of IBC.

## Conclusion

**FIGURE 8:**
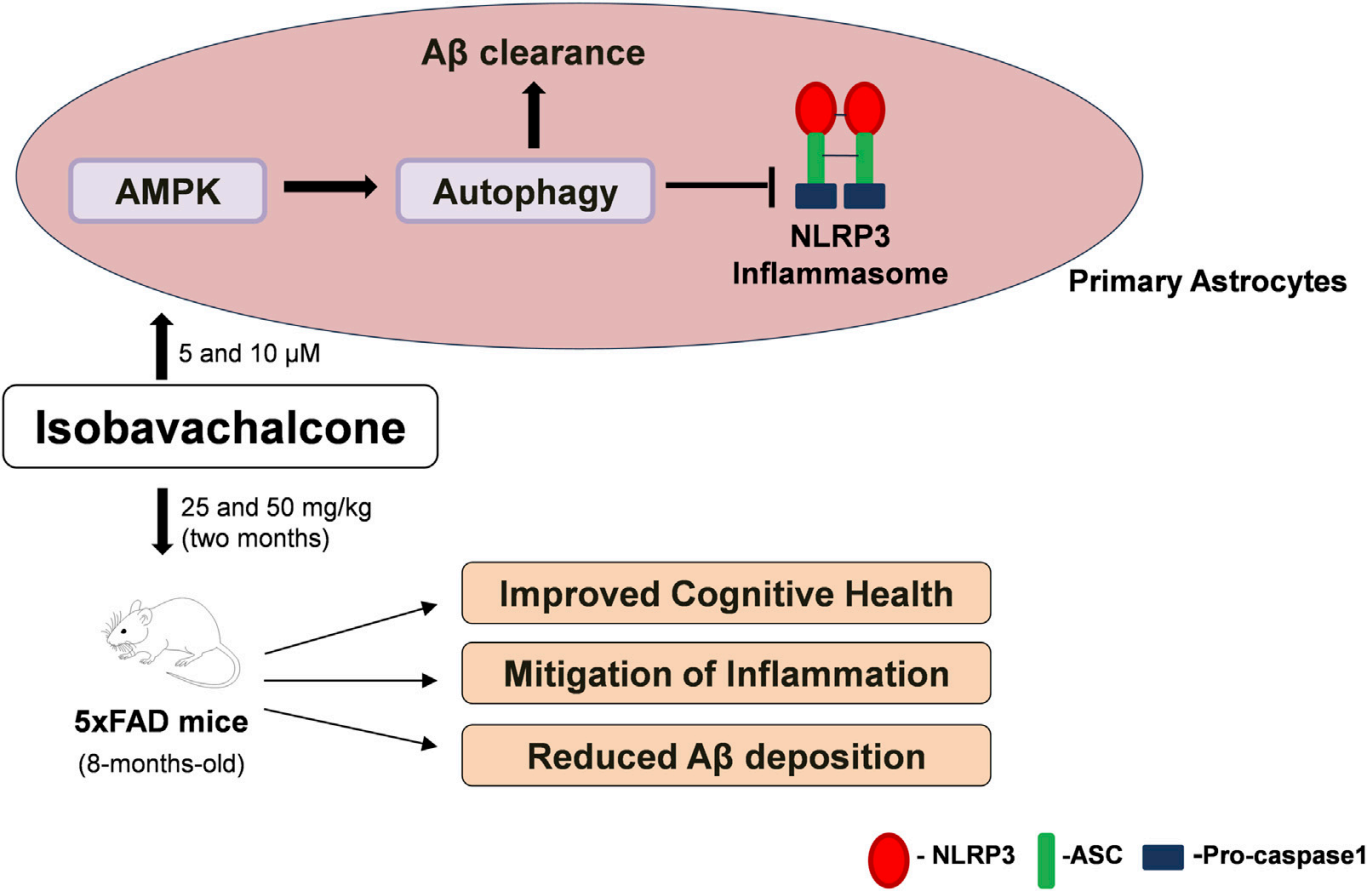
Summary of IBC activity *in-vitro* and *in-vivo*. In primary astrocytes, IBC treatment at 5 and 10 μM concentration activated AMPK and induced autophagy in cells. Induction of autophagy lead to inhibition of NLRP3 inflammasome and increased clearance of Aβ. After 2 months oral dosing of IBC in 5xFAD mice at 25 and 50 mg/kg dose, treated mice had improved cognitive health along with reduced inflammation and Aβ plaque density in mice hippocampi.

In summary, we could clear Aβ through pharmacological induction of autophagy by IBC from astrocytes and hippocampi of 5x-FAD mice. IBC, via its ability to induce AMPK-mediated autophagy, also acted as a potent inhibitor of NLRP3 inflammasome (Figure 8). This approach of pharmacologically targeting multiple disease-promoting pathways can be used for better management of AD. In this study, we used IBC to demonstrate the efficacy of this strategic approach, which can be employed in the future for developing new therapeutics against AD.

## Future statement and limitations of the study

Our preclinical analysis of IBC in 5x-FAD model warrants the translation of study in human subjects. Therefore, we are planning to conduct regulatory safety and toxicology studies to take IBC further to test its safety and efficacy in humans.

The main limitation of this study is the smaller animal cohort. Considering the dynamics of behavioral assays, larger animal groups are recommended. Since each mouse is an independent biological replicate, we have seen striking differences across the IBC-treated and untreated groups, with consistent results within groups. We believe that even when group size increases the changes observed by IBC treatment will remain significant.

## Data Availability

The original contributions presented in the study are included in the article/Supplementary Material, further inquiries can be directed to the corresponding author.

## Abbreviations

Aβ, amyloid-beta; AD, Alzheimer disease; AMPK, 5′ adenine monophosphate-activated protein kinase; ASC, apoptosis-associated speck-like protein containing a caspase recruitment domain; ATG, autophagy related; ATP, 5′ adenosine triphosphate; BCL2, B-cell lymphoma; BECN1, beclin 1; CAMKK2, calcium/calmodulin-dependent protein kinase kinase 2; CASP1, caspase-1; DAMP, damage-associated molecular patterns; DAPI, 4,6-diaminido-2-phenylindole; FIP200, Focal adhesion kinase family Interacting Protein of 200 kD; GFAP, glial fibrillary acidic protein; IBA1, ionized calcium-binding adapter molecule 1; IBC, isobavachalcone; IHC, immunohistochemistry; IL-18, interleukin-18; IL-1β, interleukin-1β; LAMP-1, lysosomal-associated membrane protein 1; LC3, light chain 3; LKB1, liver kinase B1; LPS, lipopolysaccharide; MTOR, mechanistic target of rapamycin kinase; NeuN, neuronal nuclear protein; NFT, neurofibrillary tangles; NLRP3, NOD-like receptor family pyrin domain containing 3; SQSTM1, Sequestosome 1; ULK-1, unc-51-like kinase 1
