# Supplementary material for: Isobavachalcone ameliorates Alzheimer disease pathology by autophagy-mediated clearance of amyloid beta and inhibition of NLRP3 inflammasome in primary astrocytes and 5x-FAD mice
Source: Front Pharmacol. 2025 Mar 20;16:1525364. doi: 10.3389/fphar.2025.1525364 (PMC11965660; doi:10.3389/fphar.2025.1525364)
Supplement: Supplementary file 1 [file DataSheet1.docx]

**Isobavachalcone ameliorates Alzheimer disease pathology by autophagy-mediated clearance of amyloid beta and inhibition of NLRP3 inflammasome in primary astrocytes and 5x-FAD mice**

**Supplementary files.**

**
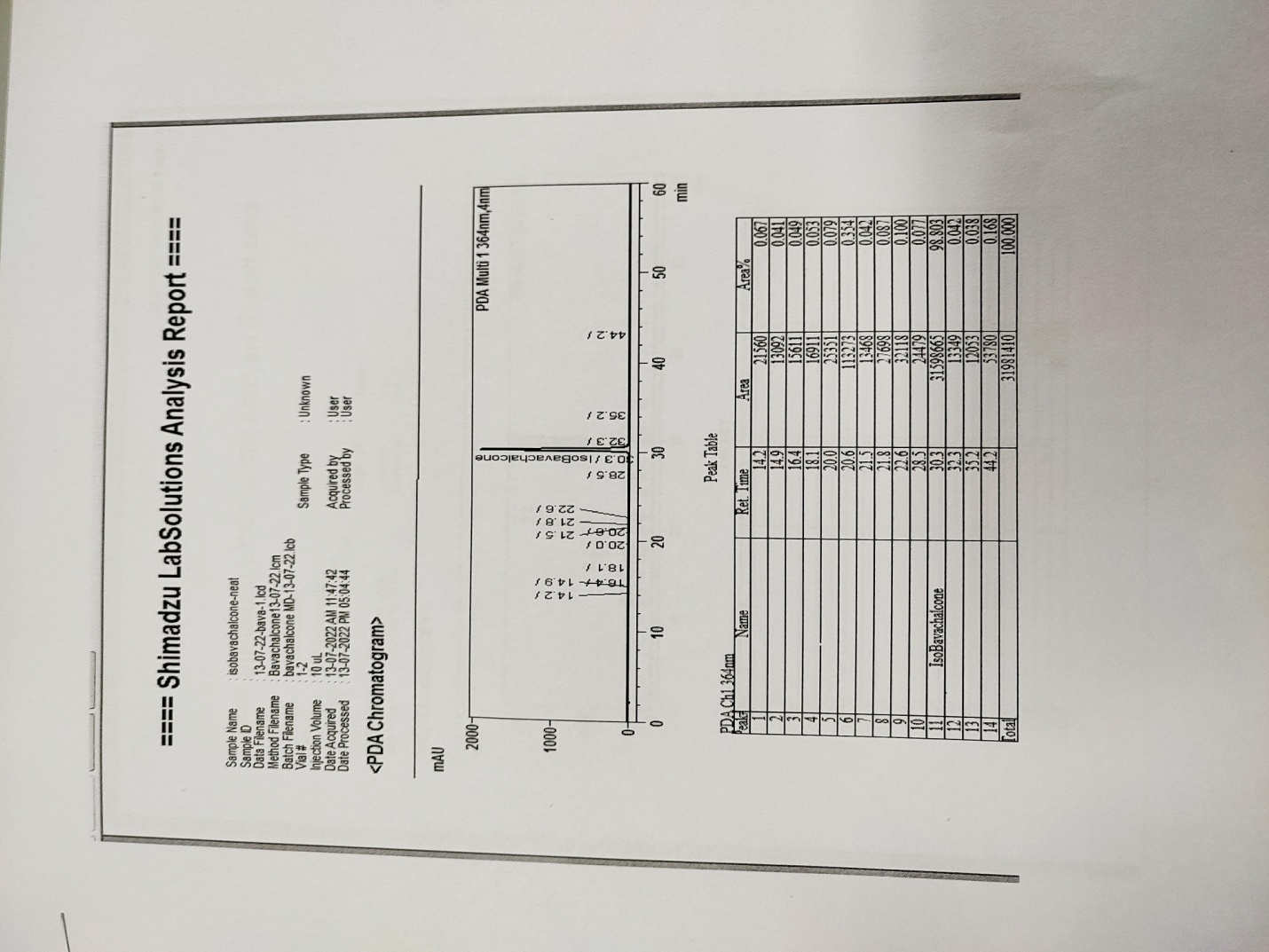
**

**Figure S1.** HPLC analysis of isolated IBC from *psoralea corylifolia* seeds showing purity of IBC as 98.8%.


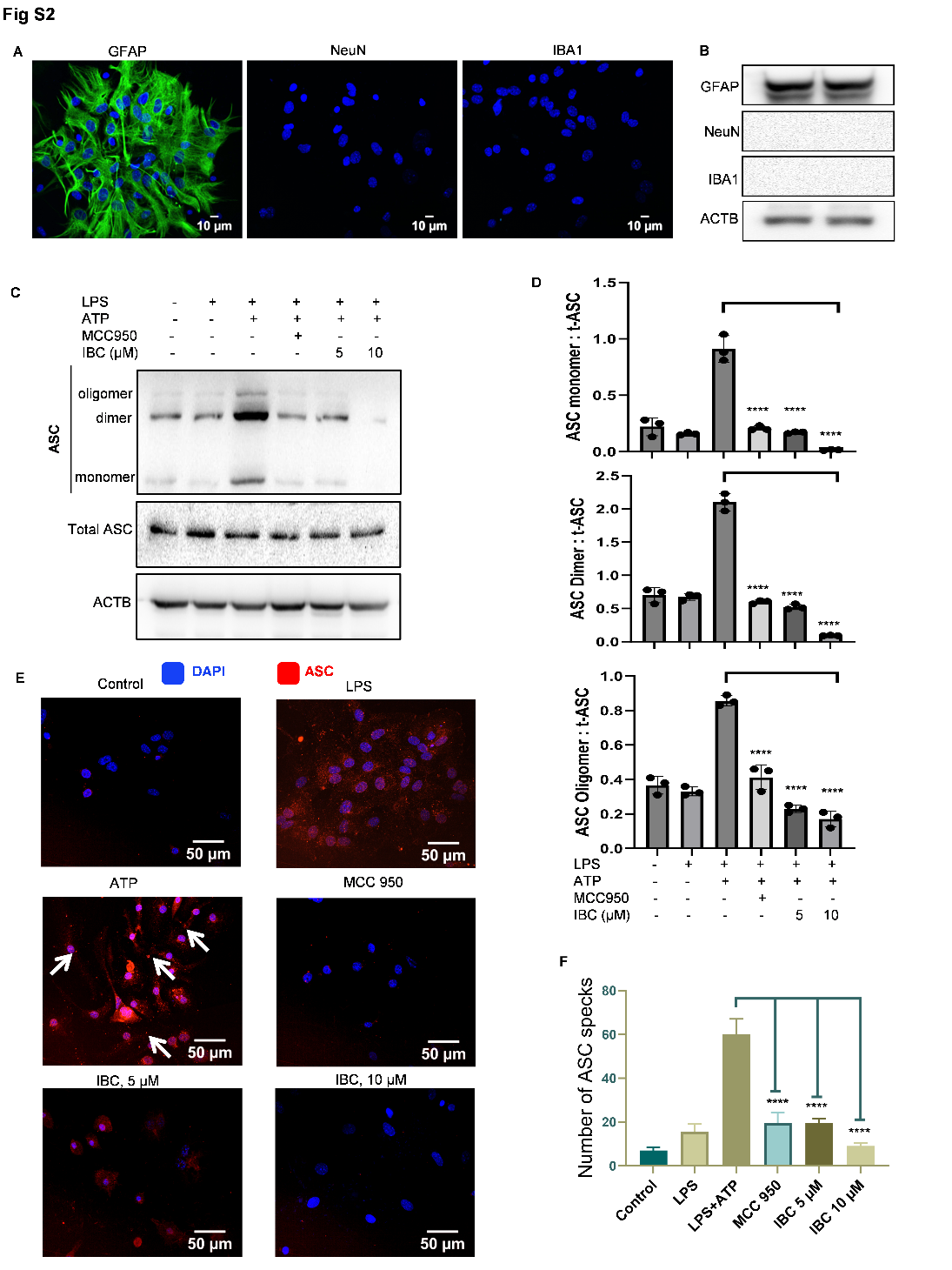


**Figure S2.** Cells were isolated from the cortices of 3-4 days old C57BL/6J mice pups and analyzed. (**A**) Immunofluorescence images depicting different brain cell populations in isolated cells where GFAP stains astrocytes, NeuN stains neurons and IBA1 stains microglial cells. Scale bars were inserted by ImageJ software. (**B**) Immunoblots of isolated primary cells. IBC suppresses ASC oligomerization. After giving treatment for NLRP3 inflammasome activation, cells were analyzed for ASC protein oligomerization. ASC oligomerization was done by cross-linking protein using suberic acid. (**C**) Immunoblots showing the effect of IBC on ASC oligomerization in primary astrocytes. (**D**) Densitometry analysis of Figure S1I, ASC monomer, ASC dimer, and ASC oligomer, normalized with total ASC protein. (**E**) Representative images of the effect of IBC (10 μM) on ASC (red) speck formation. White arrows pointing towards red dots, representing intracellular ASC specks. The scale bars were drawn by Image J software. (**F**) Graph representing the number of ASC specks in Figure S1K. Statistical significance of data was measured through one-way ANOVA and Bonferroni test. p values ****p < 0.0001, ***p < 0.001, **p < 0.01, *p < 0.05.


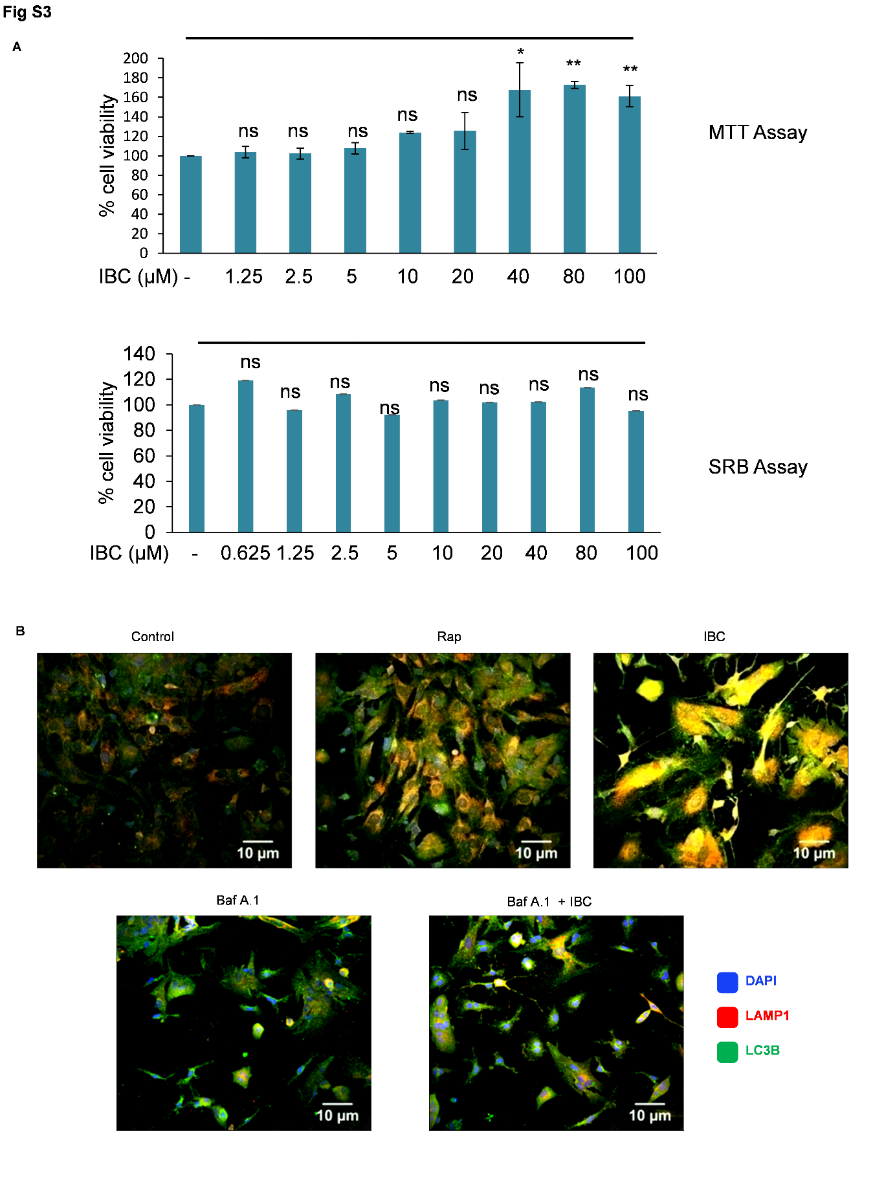


**Figure S3**. Primary astrocytes were treated with different concentration of IBC and then its safety profile was analyzed through cell viability assays. **(A)** MTT and SRB assay following 24 h treatment of IBC at different concentrations. IBC induces autophagy in primary astrocytes (**B**) Full figures of the images given in Figure 2C. DAPI (blue fluorescence), LAMP1 (red fluorescence) and LC3B (green fluorescence). Images were captured in CQ1 high throughput imaging system. Scale bars were inserted by ImageJ software. The statistical significance of data was calculated through one way ANOVA analysis, followed by Bonferroni test as post hoc. p values ***p < 0.001, **p < 0.01, *p < 0.05


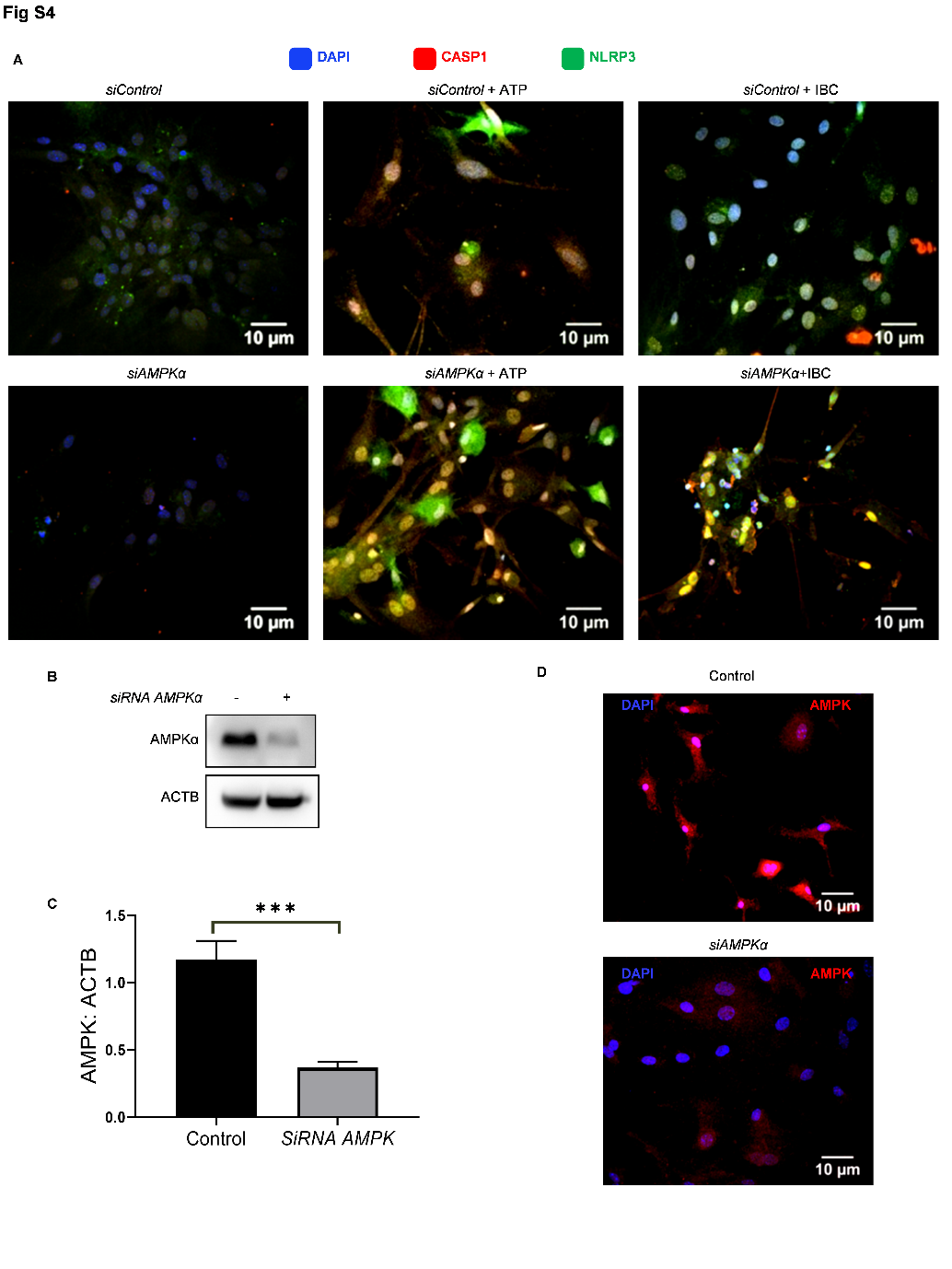


**Figure S4.** (**A**) Full images of the figures given in Figure 3F. DAPI (blue fluorescence), CASP1 (red fluorescence) and NLRP3 (green fluorescence). (**B**) AMPK expression in primary astrocytes following treatment of cells with s*iAMPK* is seen through immunoblotting. (**C**) Densitometry of Figure S3E. (**D**) Expression of AMPK protein visualized through immunofluorescence. Scale bars were drawn by ImageJ software. Data was analyzed through one-way ANOVA analysis and the Bonferroni *post hoc* test. p values ***p < 0.001.


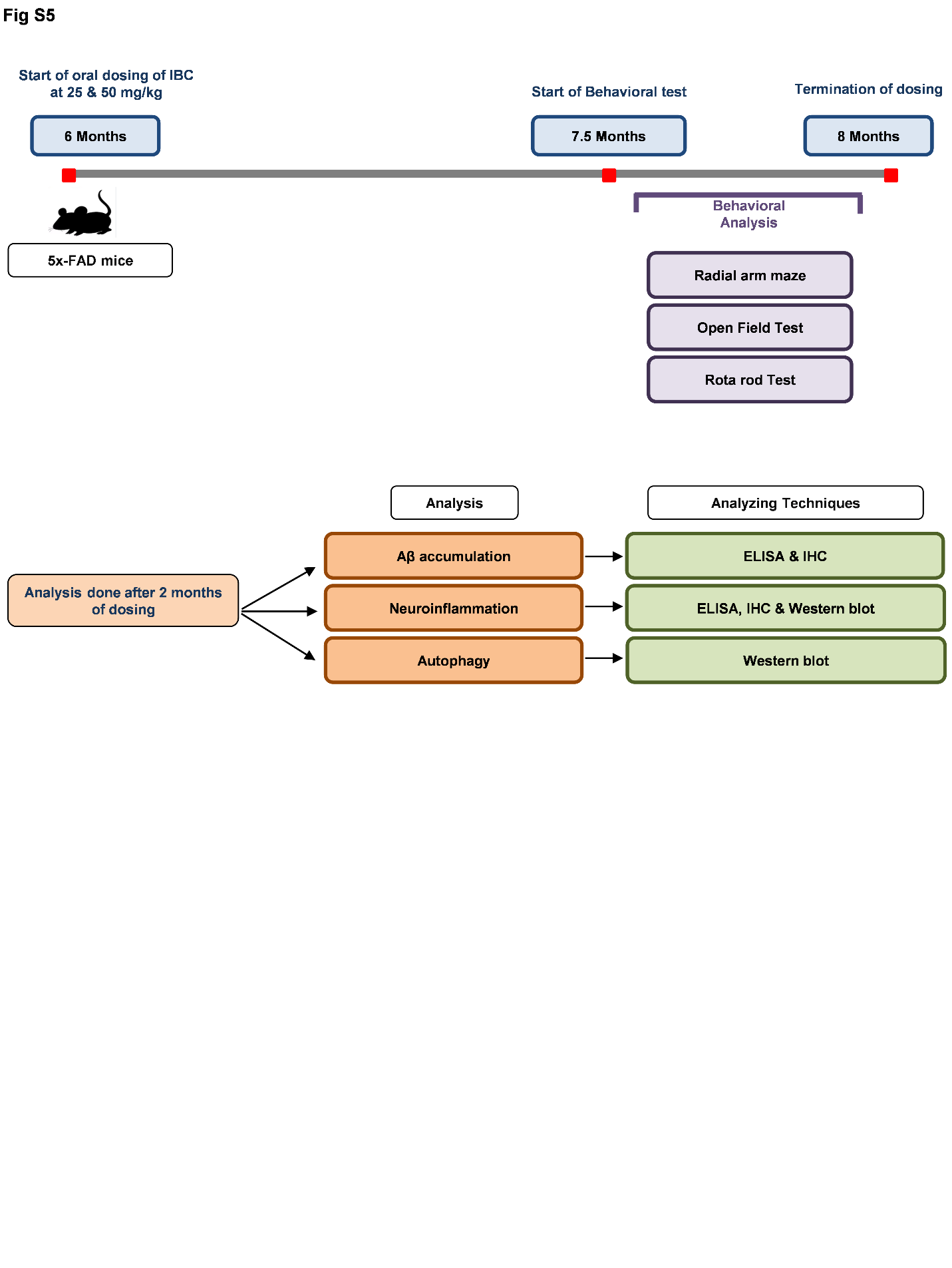
 **Figure S5.** Study plan for IBC in 5x-FAD transgenic mice. Oral dosing of IBC at 25 mg/kg and 50 mg/kg concentration was started in six months of 5x-FAD mice. After 1.5 months, behavioral experiments, radial arm maze, open field test and rotarod were initiated, which continued for two weeks. At the end of the 8^th^ month, the study was terminated by sacrificing mice and collecting blood and brain tissue. Levels of Aβ were checked through ELISA in blood plasma, and deposits were seen in the hippocampus through IHC assay. To measure the neuroinflammation, levels of IL-1β were analyzed in cortex tissue through ELISA and GFAP expression was seen in the hippocampus via IHC assay. Levels of autophagy and inflammation proteins were also analyzed in the hippocampus through immunoblotting.


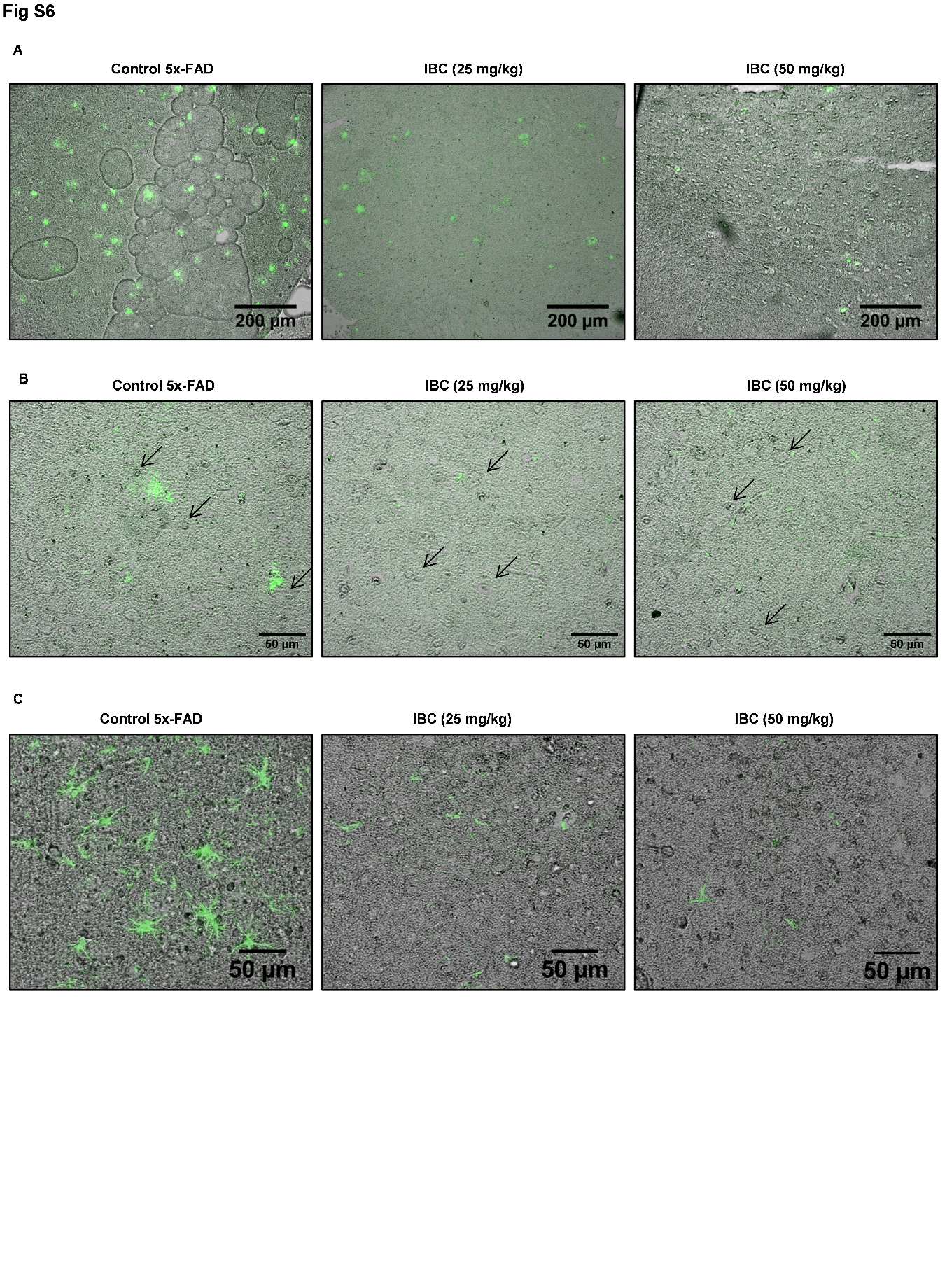
**Figure S6.** IBC alleviates Aβ deposition and neuroinflammation in 5x-FAD mice. (**A**) Fluorescent images of Figure 6A merged with brightfield images of the same tissue area. (**B**) Higher magnification images (60X) displaying Aβ deposition around pyramidal neurons (pointed by arrows) in CA region. (**C**) Fluorescent images of Figure 6E merged with brightfield images of the same tissue area. Images were acquired in CQ1 high throughput imaging system. Scale bars were inserted using ImageJ software.
